# High-pressure synthesis of ε-FeOOH from β-FeOOH and its application to the water oxidation catalyst†

**DOI:** 10.1039/d0ra09895g

**Published:** 2020-12-18

**Authors:** Kazuhiko Mukai, Tomiko M. Suzuki, Takeshi Uyama, Takamasa Nonaka, Takeshi Morikawa, Ikuya Yamada

**Affiliations:** Toyota Central Research & Development Laboratories Yokomichi 41-1 Nagakute Aichi 480-1192 Japan e1089@mosk.tytlabs.co.jp +81-561-63-6119 +81-561-71-7698; Department of Materials Science, Graduate School of Engineering, Osaka Prefecture University 1-2 Gakuen Sakai Osaka 599-8570 Japan

## Abstract

Research on materials under extreme conditions such as high pressures provides new insights into the evolution and dynamics of the earth and space sciences, but recently, this research has focused on applications as functional materials. In this contribution, we examined high-pressure/high-temperature phases of β-FeO_1−*x*_(OH)_1+*x*_Cl_*x*_ with *x* = 0.12 (β-FeOOH) and their catalytic activities of water oxidation, *i.e.*, oxygen evolution reaction (OER). Under pressures above 6 GPa and temperatures of 100–700 °C, β-FeOOH transformed into ε-FeOOH, as in the case of α-FeOOH. However, the established pressure–temperature phase diagram of β-FeOOH differs from that of α-FeOOH, probably owing to its open framework structure and partial occupation of Cl^−^ ions. The OER activities of ε-FeOOH strongly depended on the FeOOH sources, synthesis conditions, and composite electrodes. Nevertheless, one of the ε-FeOOH samples exhibited a low OER overpotential compared with α-FeOOH and its parent β-FeOOH, which are widely used as OER catalysts. Hence, ε-FeOOH is a potential candidate as a next-generation earth-abundant OER catalyst.

## Abstract 2

Research on materials under extreme conditions such as high pressures provides new insights into the evolution and dynamics of the earth and space sciences, but recently, this research has focused on applications as functional materials.

## Graphical abstract

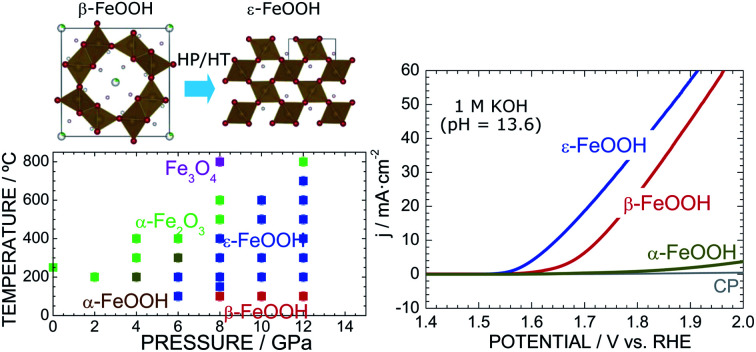

## 1 Introduction

One of the polymorphs of FeOOH, namely, β-FeOOH (akaganeite), has attracted interest in highly diverse fields. In the 20th century, it was examined in the context of the atmospheric rusting of iron and steel,^1,2^ whereas it is currently being investigated as a catalyst for water oxidation (oxygen evolution reaction; OER)^3–7^ or as a negative electrode for Li- and Na-ion batteries.^8–11^ Beside applications to functional materials, β-FeOOH has been received considerable attention in the fields of earth and space sciences and natural history because it was also discovered in the Gusev crater and the Meridiani Planum on Mars^12,13^ and in the cannon shot of the *Mary Rose*, King Henry VIII's shipwrecked flagship.^14^

**Fig. 1:**
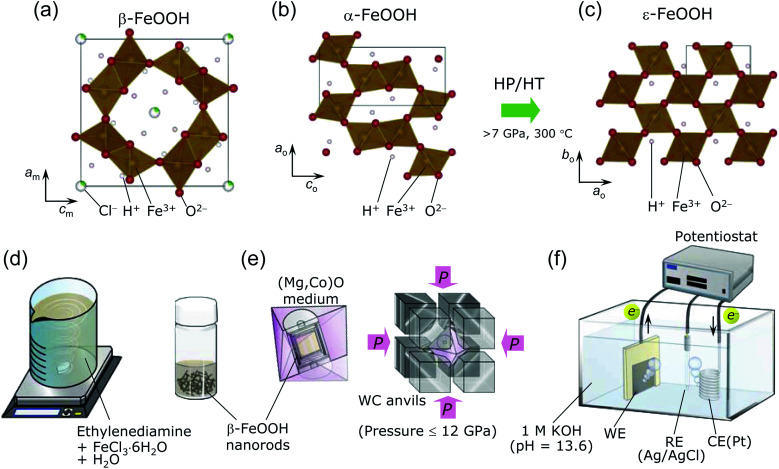
Crystal structure of (a) β-FeOOH (*I*2/*m*) with monoclinic symmetry (b) α-FeOOH (*Pnma*) with orthorhombic symmetry, and (c) ε-FeOOH (*Pmn*2_1_) with orthorhombic symmetry. The square represents the unit cell. The Cl^−^ ions partially occupy the β-FeOOH lattice to maintain the [2 × 2] tunnels. α-FeOOH transforms into ε-FeOOH under HP/HT conditions at above 7 GPa and 300 °C. Schematics of the procedures for (d) synthesizing β-FeOOH, (e) the HP/HT treatment, and (f) evaluating the OER activity.

β-FeOOH was generally synthesized by hydrolyzing FeCl_3_ solutions at 60–100 °C,^1,15–20^ before it was discovered as a natural product in the Akagane metal mine, Japan.^21^Fig. 1a shows the crystal structure of β-FeOOH, in which FeO_6_ octahedra form [2 × 2] tunnels *via* double corner-linked bands.^22–26^ A certain number of guest Cl^−^ ions are necessary to maintain the [2 × 2] tunnels, leading to the specific chemical formula FeO_1−*x*_(OH)_1+*x*_Cl_*x*_.^1,2,16^ Its structure was initially proposed to have tetragonal symmetry with the *I*4/*m* space group,^22–24^ but recent X-ray diffraction (XRD) measurements revealed monoclinic symmetry with the *I*2/*m* space group.^25,26^ The monoclinic distortion is extremely small, as understood by its lattice parameters: *a*_m_ = 10.5536(7) Å, *b*_m_ = 3.03449(8) Å, *c*_m_ = 10.5740(4) Å, and *β* = 90.086(5)°.^26^ β-FeOOH is isostructural to α-MnO_2_, which contains Na^+^ (manjiroite), K^+^ (cryptomelane), Ba^2+^ (hollandite), or Pb^2+^ (coronadite) ions as the guest in the [2 × 2] tunnels.^27^ A series of α-MnO_2_ has also been employed as the catalyst for the OER^28–30^ and as the electrode for nonaqueous or aqueous secondary batteries.^31,32^

Many studies have been performed on β-FeOOH, but mostly under ambient pressure. However, a high-pressure/high-temperature (HP/HT) study could provide insights into its fundamental structural and electronic properties to gain an in-depth understanding of the evolution and dynamics of earth and solar system. Indeed, in the case of α-FeOOH, a pressure-induced spin state transition (t^3^_2g_e^2^_g_ → t^5^_2g_) and hydrogen bond symmetrization occur at pressures exceeding 40 GPa.^33–38^ Moreover, α-FeOOH transforms into ε-FeOOH under HP/HT conditions at above 7 GPa and 300 °C.^39–41^Fig. 1b and c show the crystal structures of α-FeOOH^47^ and ε-FeOOH,^39–41^ respectively, wherein the latter adopts a distorted rutile structure with the *Pmn*2_1_ space group, forming [1 × 1] tunnels. Among the three FeOOH polymorphs, β-FeOOH is significantly less compressible as a function of pressure (at room temperature), owing to its high bulk modulus of 284(1) GPa.^42^ The HP/HT phase of β-FeOOH is currently unknown, although Xu *et al.*^43^ reported that ε-FeOOH is a HP phase of β-FeOOH without any experimental evidence.

Regarding the OER catalyst, materials synthesized from β-FeOOH under HP/HT conditions could offer a wide range of material options to enhance OER performance, because current studies on the FeOOH polymorphs focus on fabricating composite electrodes or heterodoping.^3–7,44–46^ Furthermore, earth-abundant catalysts such as Fe-based compounds are required to realize the global scalability within the framework of sustainable energy technologies. Note that the OER activities of the HP/HT phases of β-FeOOH and ε-FeOOH have not been reported thus far, including theoretical calculations on FeOOH polymorphs.^48,49^ The moderate strength of metal bonds (Fe–O bonds in this case) is generally accepted to play a crucial role in the OER performance.^7^ Furthermore, the O–H⋯O configuration is highly asymmetric under ambient pressure; for instance, the O–H and H⋯O lengths are 0.88 and 1.94 Å, respectively, for α-FeOOH^47^ and 0.86–0.98 and 2.10–2.49 Å, respectively, for β-FeOOH.^26^ Although β-FeOOH exhibited the maximum bulk modulus,^42^ the strength of the Fe–O bond and the asymmetric O–H⋯O configuration should be altered under HP/HT conditions and should significantly affect the OER performance of β-FeOOH. In the case of the quadruple perovskite oxide CaCu_3_Fe_4_O_12_, which was synthesized under HP/HT conditions, the OER performance was enhanced when the interatomic distance between the nearest neighboring OH adsorbates was *ca.* 2.6 Å.^50^

In this contribution, we revealed HP/HT phases of β-FeOOH under pressures of 2–12 GPa and temperatures below 800 °C, as well as the OER activity of the obtained materials. Several methods such as synchrotron XRD measurements, X-ray absorption spectroscopy (XAS), and ^57^Fe Mössbauer spectroscopy were employed to clarify their structural and electronic properties. Consequently, β-FeOOH was found to transform into ε-FeOOH along a different pressure–temperature phase diagram from that of α-FeOOH. In addition, we found that the ε-FeOOH sample prepared at 8 GPa and 400 °C exhibited a lower OER overpotential than those of the parent β-FeOOH and α-FeOOH.

## 2 Experimental

### 2.1 Preparation of β-FeOOH

β-FeOOH is usually prepared by aging solutions at 60–100 °C for several days, offering 100–500 nm-size particles.^1,15,18,20^ We, however, used a recently developed method, namely, a combination of spontaneous peptization and Ostwald ripening, to obtain β-FeOOH colloidal solution at room temperature (*ca.* 25 °C).^3–5^ This method provides highly crystalline and uniform β-FeOOH nanorods with diameters of 3 nm and lengths of 15 nm,^3–5^ owing to the typical advantages of the Ostwald ripening method.^51^ As shown in Fig. 1d, a colloidal solution was prepared by mixing 500 mL of an aqueous solution consisting of 0.1 M FeCl_3_·6H_2_O and ethylenediamine (CH_2_)_2_(NH_2_)_2_ (FUJIFILM Wako Pure Chemical). The pH of this mixture was maintained at 2.0–2.4. Then the solution was continuously stirred for 30 min and finally aged overnight at *ca.* 25 °C.

A sample of the β-FeOOH nanorods was removed from the colloidal solution before the HP/HT treatment. The remaining colloidal solution was dried in a Petri dish at *ca.* 25 °C and rinsed with a 0.1 M KOH (FUJIFILM Wako Pure Chemical) solution. This powder was filtered with a cellulose acetate membrane with a pore size of 0.2 μm (Toyo Roshi Kaisha) and washed three times with distilled water. The powder was finally dried at 45 °C in vacuum for 8 h. The amount of Cl^−^ ions, *x* in FeO_1−*x*_(OH)_1+*x*_Cl_*x*_, was examined with a combination of inductively coupled plasma-atomic emission spectroscopy (ICP-AES, PS3520VDDII, Hitachi High-Technologies), and ion chromatography (IC, Dionex ICS-1500, Thermo Fisher Scientific). The ICP-AES and IC methods revealed the atomic ratios of Fe and Cl in the sample, respectively. We recorded three independent measurements to confirm the reproducibility of the *x* value.

### 2.2 HP/HT synthesis

The pristine β-FeOOH sample was heated below 800 °C and under pressures of 2–12 GPa using Walker-type equipment at Osaka Prefecture University.^50,52–54^ Approximately 40 mg of the sample was sealed into a Pt capsule, and then packed into an octahedral (Mg,Co)O pressure medium with one side length of 14 mm (Mino Ceramics), together with a BN sleeve (Denka) and a graphite heater (Mechanical Carbon Industry). The (Mg,Co)O octahedron was placed at the corners of eight WC anvils (TF06, Fuji Die) and compressed at *ca.* 25 °C and 2, 4, 6, 8, 10, or 12 GPa (see Fig. 1e). After reaching the desired pressure, each sample was heated at 100, 150, 200, 300, 400, 500, 600, 700, or 800 °C for 30 min. The time approaching each target temperature was 15 min. The WC anvils were quenched to *ca.* 25 °C and then slowly decompressed to ambient pressure. Moreover, we synthesized two ε-FeOOH samples using an α-FeOOH source (denoted as pristine α-FeOOH; Koujyundo Chemical Laboratory) to compare the OER activities of α- and β-FeOOH sources. The applied pressure was 10 GPa, and the heating temperature was 400 or 500 °C. Table 1 summarizes the conditions for HP/HT synthesis, such as FeOOH source, pressure, and temperature. Hereafter, the HP/HT-treated samples are represented by their synthesis conditions in the format “sample *XX-YY*”, where *XX* and *YY* correspond to the applied pressure and heating temperature, respectively. For example, sample 2-200 was treated with a pressure 2 GPa and 200 °C. The two samples synthesized with the α-FeOOH source are labeled with the prefix α, *e.g.*, α-10-400 and α-10-500.

**Table 1:** Synthesis conditions (source, pressure, and temperature), main phase, and OER activity^a^

Sample no.	Sample notation	FeOOH source	Pressure/GPa	Temperature/°C	Main phase	*E*_*j*=1 mA cm^−2^_ a/V *vs.* RHE
1	Pristine β-FeOOH	β	—	—	β-FeOOH	1.62
2	2-200		2	200	α-Fe_2_O_3_	
3	4-200		4	200	α-FeOOH	
4	4-300		4	300	α-Fe_2_O_3_	
5	4-400		4	400	α-Fe_2_O_3_	
6	6-100		6	100	ε-FeOOH	
7	6-200		6	200	ε-FeOOH	
8	6-300		6	300	α-FeOOH	
9	6-400		6	400	α-Fe_2_O_3_	
10	8-100		8	100	β-FeOOH	
11	8-150		8	150	ε-FeOOH	
12	8-200		8	200	ε-FeOOH	1.76
13	8-300		8	300	ε-FeOOH	
14	8-400		8	400	ε-FeOOH	1.56
15	8-500		8	500	α-Fe_2_O_3_	
16	8-600		8	600	α-Fe_2_O_3_	
17	8-800		8	800	Fe_3_O_4_	
18	10-100		10	100	β-FeOOH	
19	10-200		10	200	ε-FeOOH	
20	10-300		10	300	ε-FeOOH	
21	10-500		10	500	ε-FeOOH	
22	10-600		10	600	ε-FeOOH	
23	12-100		12	100	β-FeOOH	
24	12-200		12	200	ε-FeOOH	
25	12-300		12	300	ε-FeOOH	
26	12-400		12	400	ε-FeOOH	
27	12-500		12	500	ε-FeOOH	1.62
28	12-600		12	600	ε-FeOOH	
29	12-700		12	700	ε-FeOOH	
30	12-800		12	800	α-Fe_2_O_3_	
31	Pristine α-FeOOH	α	—	—	α-FeOOH	1.82
32	α-10-400		10	400	ε-FeOOH	1.64
33	α-10-500		10	500	ε-FeOOH	1.61

a The potential *vs.* reversible hydrogen electrode (RHE) at which the anodic current density (*j*) was 1 mA cm^−2^.

### 2.3 Characterization

The crystal structures were examined with powder XRD using Fe-Kα radiation (D8 ADVANCE, Bruker AXS) and synchrotron radiation sources. The synchrotron XRD patterns were measured at the BL5S2 beamline at the Aichi Synchrotron Radiation Center using a two-dimensional (2D) detector, PILATUS 100K (Dectris). Each sample was packed into a borosilicate glass capillary with a diameter of 0.3 mm (WJM-Glas) and then exposed to X-rays for 20 min to acquire XRD data over a wide 2*θ* range.^55^ The wavelength of the X-rays was determined to be 0.799436(2) Å using standard silicon powder (NIST 640d). Rietveld analyses were performed using RIETAN-FP software,^56^ and crystal structures were drawn with the VESTA software.^57^

The local structures and electronic states of pristine β-FeOOH and sample 8-400 were investigated by XAS at the Toyota beamline (BL33XU) at SPring-8. XAS was also performed on Fe metal (thickness = 10 μm, Nilaco) and α-Fe_2_O_3_ (FUJIFILM Wako Pure Chemical) as a reference. Approximately 3 mg of α-Fe_2_O_3_ and BN powder (FUJIFILM Wako Pure Chemical) were mixed with a mortar and pestle and then pressed into a pellet with a diameter of 10 mm and a thickness of *ca.* 0.2 mm. XAS data were recorded at *ca.* 25 °C in transmission mode. Fe K-edge X-ray absorption near-edge structure (XANES) and the Fourier transform of the *k*^3^-weighted Fe K-edge extended X-ray absorption fine structure (EXAFS) spectra were analyzed using ATHENA and ARTHEMIS software.^58^ The experimental setup for XAS and spectral analyses are described in further detail elsewhere.^4,59^

We performed Mössbauer spectroscopy on pristine β-FeOOH and samples 8-400 and α-10-400 to examine the valence state and local environment of their respective Fe ions. Pristine β-FeOOH and sample 8-400 were measured (without a magnetic field) at *ca.* 25 °C and liquid-nitrogen temperature (*ca.* −196 °C), whereas sample α-10-400 was examined only at *ca.* 25 °C. The radiation source was a ^57^Co/Rh matrix with a radioactivity of 370 MBq for pristine β-FeOOH and sample 8-400, while 1.85 GBq for sample α-10-400. A multi-channel-analyzer with 512 channels (CMCA-550, Wissenschaftliche Elektronik, or MCS-plus, Ortec) was employed to acquire spectral data. One channel was determined to be 0.055598 mm s^−1^ for pristine β-FeOOH and sample 8-400, while 0.0791871 mm s^−1^ for sample α-10-400, by calibrating the Doppler velocity with Fe foil. Spectral analyses were conducted using MossWinn 4.0i software.^60^

Thermogravimetric (TG; Thermo plus EVO2, TG8120, Rigaku) analyses were conducted for pristine β-FeOOH and samples 8-100 through 8-500, to clarify changes in *x* upon heating and the thermal stabilities of β-FeOOH and ε-FeOOH. TG curves were recorded with a heating rate of 20 °C min^−1^ up to 1000 °C under flowing air (100 mL min^−1^). After the TG analysis, XRD measurements were performed using an Fe-Kα radiation source (D8 ADVANCE, Bruker AXS).

Particles sizes and morphologies were examined in two different types of scanning electron microscope (SEM), that is, a conventional system with a tungsten filament (S-3600 N, Hitachi High-Technologies) and a field-emission (FE) system using an emitter tip (SU8020, Hitachi High-Technologies). For the conventional system, 1 mg of sample, which was attached to carbon tape, was coated with electrically conductive Au particles (IB-3, Eiko). Meanwhile, for the FE system, samples were coated with Os particles (HPC-1S, Vacuum Device). The accelerating voltages were 15 and 1 kV for the conventional and FE-SEM, respectively. Particle distribution and average size (*d*_ave_) were determined by using the ImageJ software.^61^

We also investigated the distribution of Cl^−^ ions in the particles of pristine β-FeOOH and sample 8-400 with an energy dispersive X-ray (EDX) spectrometer (Ultim Extreme, Oxford Instruments). The specimens were prepared through ion (Ar) milling under an accelerating voltage of 6 kV at −160 °C (Model685 PECS II, Gatan). We recorded FE-SEM images using Everhart–Thornley-type detector and backscattered electron (BSE) images using an in-lens detector (Ultra55, Carl Zeiss). The accelerating voltage was 5 or 7 kV.

### 2.4 Evaluation of OER activity

We examined the OER activity of seven samples in an Ar-purged 1 M KOH solution (pH = 13.6) with a three-electrode configuration (see Fig. 1f);^3–5^*i.e.*, pristine β-FeOOH, samples 8-200, 8-400, 12-500, pristine α-FeOOH, and samples α-10-400 and α-10-500. We chose these samples considering each crystal phase, crystallinity, particle size/morphology, and the FeOOH source employed. Approximately 0.3–0.5 mg of each sample was attached to carbon paper (CP; TGP-H-060, TORAY) with a geometrical surface area of 1 cm^2^ (1 cm × 1 cm) and used as a working electrode (WE). This procedure is slightly different from the method previously reported,^3–5,48^ where a colloidal solution of β-FeOOH nanorods was injected into CP. The total surface area of the electrode was 2 cm^2^ by adding both the front and back sides. The counter electrode (CE) was a Pt wire, whereas the reference electrode (RE) was Ag/AgCl. Linear sweep voltammetry (LSV; ALS612E, ALS) was performed at a scan rate of 5 mV s^−1^, and chronoamperometry was conducted at +0.7 V *vs.* Ag/AgCl for 1800 seconds to clarify the stability of the anodic currents. Electrochemical impedance spectroscopy (EIS, CHI-614C, ALS) was performed on pristine β-FeOOH and samples 8-200, 8-400, and 12-500 in the frequency range from 0.1 to 10^5^ Hz under flowing Ar (20 mL min^−1^).

## 3 Results and discussion

### 3.1 Crystal structure and phase diagram

**Fig. 2:**
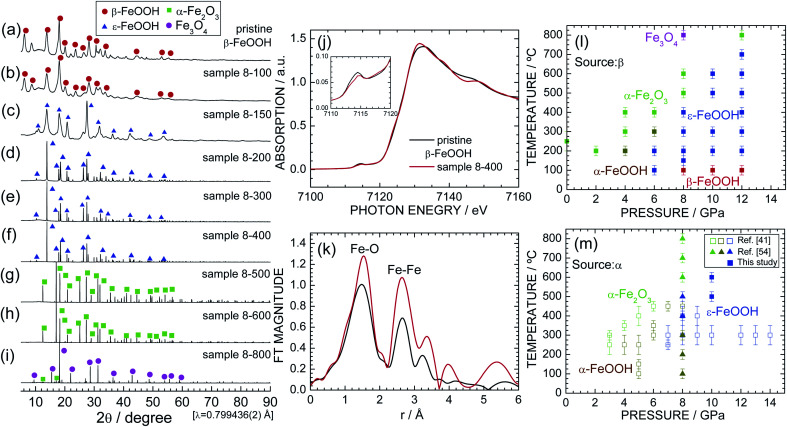
Synchrotron XRD patterns of samples (a) pristine β-FeOOH, (b) 8-100, (c) 8-150, (d) 8-200, (e) 8-300, (f) 8-400, (g) 8-500, (h) 8-600, and (i) 8-800. Diffraction lines from the β-FeOOH, ε-FeOOH, α-Fe_2_O_3_, and Fe_3_O_4_ phases are indicated by red circles, blue triangles, green squares, and purple circles, respectively. (j) Fe K-edge XANES and (k) the Fourier transforms of *k*^3^-weighted Fe K-edge EXAFS spectra of pristine β-FeOOH and sample 8-400. The pre-edge peak at *ca.* 7115 eV is enlarged in the inset. Pressure–temperature phase diagram for the (l) β-FeOOH and (m) α-FeOOH sources. Data from ref. 41 and 54 are also shown in (m) for comparison.

Fig. 2a–i show the synchrotron XRD patterns of pristine β-FeOOH and samples 8-100 through 8-800. The XRD pattern of pristine β-FeOOH was assigned to the typical crystal structure of β-FeOOH.^22–26^ We first analyzed the data using the tetragonal symmetry with *I*4/*m* space group,^26^ providing reliability indices of *R*_wp_ = 5.99% and *S* = 1.09. However, employing the monoclinic symmetry with *I*2/*m* space group improved these reliability indices to *R*_wp_ = 4.11% and *S* = 0.74. The tetragonal symmetry only has one Fe site, whereas two Fe sites exist in monoclinic symmetry. As described later, the presence of two Fe sites was confirmed by Mössbauer spectroscopy. The lattice parameters were determined to be *a*_m_ = 10.4758(20) Å, *b*_m_ = 3.0282(2) Å, *c*_m_ = 10.5427(22) Å, and *β*_m_ = 89.51(2) ° by ignoring the presence of H^+^ ions. The results of the Rietveld analysis are summarized in Fig. S1† and Table 2. The nearest bond distances between Fe and O ions (*d*_Fe–O_) were calculated to be 1.867(13) Å × 1, 2.175(14) Å × 1, 1.948(6) Å × 2, and 2.242(7) Å × 2, whereas that between Fe and Fe ions (*d*_Fe–Fe_) was 3.028(1) Å. The lattice parameters were similar to previously reported values,^22–24^ but two of the six *d*_Fe–O_ values [1.867(13) and 2.175(14) Å] slightly differ from those in the literature (1.90 and 2.05 Å).^26^ The relatively variable *d*_Fe–O_ value probably originated from the low aspect ratio of pristine β-FeOOH; *i.e.*, the diameter and length are 3 and 15 nm, respectively.^3–5^

**Table 2:** Structural parameters of pristine β-FeOOH determined by the Rietveld analysis^b^

Atom	Wyckoff position	Occupancy^a^	*x*	*y*	*z*	*B*_iso_ (Å^2^)
Fe1	4*i*	1	0.858(1)	0	0.338(1)	0.9(1)
Fe2	4*i*	1	0.345(1)	0	0.143(1)	0.9(1)
O1	4*i*	1	0.661(1)	0	0.276(1)	0.4(1)
O2	4*i*	1	0.688(1)	0	0.012(1)	0.4(1)
O3	4*i*	1	0.303(1)	0	0.377(1)	0.4(1)
O4	4*i*	1	0.036(1)	0	0.350(1)	1.2(1)
Cl1	2*a*	0.12	0	0	0	0.3(1)

a We fixed site occupancies to 1 or 0.12.

b Space group: *I*2/*m*, *a*_m_ = 10.4758(20) Å, *b*_m_ = 3.0282(2) Å, *c*_m_ = 10.5427(22) Å, *β*_m_ = 89.51(2) °, *R*_wp_ = 4.11%, and *S* = 0.74.

Sample 8-100 also maintained the β-FeOOH-type structure, whereas sample 8-150 possessed a different crystal structure from pristine β-FeOOH. After careful examination, the crystal structure of sample 8-150 was assigned to the orthorhombic symmetry with the *Pmn*2_1_ space group, *i.e.*, an ε-FeOOH-type structure. The Rietveld analysis revealed orthorhombic lattice parameters of *a*_o_ = 2.9688(7) Å, *b*_o_ = 4.4558(10) Å, and *c*_o_ = 5.0077(12) Å (not shown). The XRD pattern of 8-150 exhibits broad and weak diffraction lines, indicating low crystallinity and/or a small crystallite size. By contrast, sample 8-200 shows sharp and strong diffraction lines with the ε-FeOOH-type structure (Fig. 2d). Further, ε-FeOOH was maintained up to 400 °C, but further increasing the heating temperature caused other structural transitions. Specifically, sample 8-600 was an α-Fe_2_O_3_ (hematite) phase, and sample 8-800 consisted of α-Fe_2_O_3_ and Fe_3_O_4_ (magnetite) phases, wherein the weight fraction of the latter phase was estimated to be 92.8%. The main phase for each sample is summarized in Table 1. The formation of α-Fe_2_O_3_ is represented by

1 2FeOOH → α-Fe_2_O_3_ + H_2_O↑,

whereas the formation of Fe_3_O_4_ is represented by

2 6α-Fe_2_O_3_ → 4Fe_3_O_4_ + O_2_↑.

The weight losses (Δ*w*) were calculated to be −10.14% for eqn (1) and −3.34% for eqn (2). The formation of Fe_3_O_4_ significantly differs from the situation under ambient pressure, where α-Fe_2_O_3_ remains stable up to 1000 °C.^1,16,21,22^

Fig. S2† and Table 3 summarize the Rietveld analysis results of sample 8-400 as an example. The *a*_o_, *b*_o_, and *c*_o_ values were determined to be 3.0020(1), 4.4570(1), and 4.9527(1) Å, respectively, which slightly differ from those for sample 8-150 owing to differences in the crystallinity. The *d*_Fe–O_ values were calculated to be 1.942(3) Å × 1, 2.137 Å × 1, 1.970(1) Å × 2, and 2.097(1) Å × 2, whereas *d*_Fe–Fe_ was 3.002(1) Å. These structural parameters are comparable with those for previous ε-FeOOH compounds that were synthesized from an α-FeOOH source.^40,41,54^ Based on the geometry of polyhedral distortions, the distortion index in the FeO_6_ octahedron [DI(FeO_6_)] is defined as^62^3
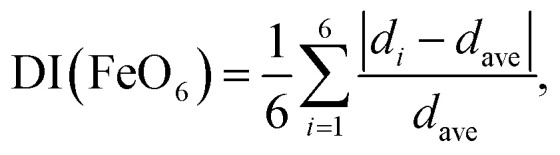
where *d*_*i*_ and *d*_ave_ are the *i*^th^ and average *d*_Fe–O_, respectively. Because the DI(FeO_6_) values for pristine β-FeOOH and sample 8-400 (ε-FeOOH) were calculated to be 0.100 and 0.037, respectively, the HP/HT environment evidently decreased the distortion in the FeO_6_ octahedron. Similar results were obtained for rhombohedral-structured LiCo_0.64_Mn_0.36_O_2_ (ref. 52) and orthorhombic-structured LiMnO_2_.^63^

**Table 3:** Structural parameters of sample 8-400 determined by the Rietveld analysis^b^

Atom	Wyckoff position	Occupancy^a^	*x*	*y*	*z*	*B*_iso_ (Å^2^)
Fe1	2*a*	1	0	0.220(1)	0.000(1)	0.2(1)
O1	2*a*	1	0	0.006(1)	0.342(1)	0.2(1)
O2	2*a*	1	0	0.495(1)	0.646(1)	0.2(1)

a We fixed site occupancies to 1.

b Space group: *Pmn*2_1_, *a*_o_ = 3.0020(1) Å, *b*_o_ = 4.4570(1) Å, *c*_o_ = 4.9527(1) Å, *R*_wp_ = 6.91%, and *S* = 0.63.

To clarify changes in the local structure during the β-FeOOH → ε-FeOOH transition, Fig. 2j and k show the Fe K-edge XANES and Fourier transforms of the *k*^3^-weighted Fe K-edge EXAFS spectra of pristine β-FeOOH and sample 8-400. For both samples, a pre-edge peak at *ca.* 7115 eV and a main edge peak at *ca.* 7131 eV appear in the XANES spectra. The pre-edge peak is a mixture of an electric quadrupole transition and an electric-dipole-allowed 1s → 4p transition, which is formally electric dipole forbidden.^64^ The presence of the pre-edge peak corresponds to a noncentrosymmetric geometry for the FeO_6_ octahedron, *i.e.*, a distortion of the FeO_6_ octahedron, as evident from the DI(FeO_6_) values. As understood by the Fe K-edge XANES spectrum of α-Fe_2_O_3_ (Fig. S3†), the photon energy of the main edge peak is located at 7131 eV. Hence, Fe ions were in the trivalent state for both pristine β-FeOOH and sample 8-400.

Two major peaks appear in the EXAFS spectra centered at phase-uncorrected radial distances (*r*) of approximately 1.5 and 2.7 Å, which were assigned to *d*_Fe–O_ and *d*_Fe–Fe_, respectively. More specifically, *d*_Fe–O_ = 1.48(1) Å and *d*_Fe–Fe_ = 2.66(1) Å, for pristine β-FeOOH, whereas *d*_Fe–O_ = 1.54(1) Å and 2.65(1) Å for sample 8-400. The EXAFS spectrum of pristine β-FeOOH is similar to the spectrum that we recently reported for β-FeOOH,^3–5^ although to the best of our knowledge, the EXAFS spectrum of ε-FeOOH has never been reported thus far.

To establish the pressure–temperature phase diagram for β-FeOOH, synchrotron XRD patterns of samples of 2-200–6-400, 10-100–10-600, and 12-100–12-800 are shown in Fig. S4, S5, and S6,† respectively. Based on these results, the pressure–temperature phase diagram of β-FeOOH is illustrated in Fig. 2l. The α-Fe_2_O_3_ or α-FeOOH phase exists at pressures below 4 GPa, whereas the ε-FeOOH phase forms only at pressures above 6 GPa. Surprisingly at 6 GPa, the α-FeOOH phase appears at 300 °C after the formation of ε-FeOOH below 200 °C. Thus at this pressure, the structural phase transition with temperature becomes β-FeOOH → ε-FeOOH → α-FeOOH → α-Fe_2_O_3_, although α-FeOOH was synthesized by β-FeOOH, not ε-FeOOH. At 12 GPa, ε-FeOOH appeared up to 700 °C, providing the general trend that higher pressures above 6 GPa stabilize the ε-FeOOH phase to higher temperatures above 200 °C. On the other hand at pressures above 8 GPa, the β-FeOOH phase is obtained below 100 °C. Mixtures were rarely observed, and each sample comprised an almost single phase of α-FeOOH, α-Fe_2_O_3_, Fe_3_O_4_, ε-FeOOH, or β-FeOOH.

Fig. 2m shows the pressure–temperature phase diagram for α-FeOOH for comparison, which includes data from previous HP/HT studies on α-FeOOH.^41,54^ Fig. S7† shows synchrotron XRD patterns of samples of pristine α-FeOOH, α-10-400 and α-10-500; among them, the latter two samples are single phases of ε-FeOOH. Obviously, the ε-FeOOH phase forms in the region of lower pressures (*ca.* 1 GPa) and temperatures (*ca.* 200 °C) in the case of β-FeOOH. As mentioned above, the bulk modulus of β-FeOOH [=284(1) GPa] at room temperature is the highest among the FeOOH polymorphs.^42^ Considering this result, the required pressure for the transition to ε-FeOOH would be higher in the β-FeOOH, but the obtained fact was contrary to the expectation. As illustrated in Fig. 1a, β-FeOOH possesses the open framework structure with the partial occupation of Cl^−^ ions. The lower pressure-induced transition indicates a fragility of β-FeOOH at high temperatures above 100 °C. Indeed, β-FeOOH was reported to decompose into α-Fe_2_O_3_ and H_2_O at lower temperatures (under ambient pressure) compared to α-FeOOH, due to its strengthening of the hydrogen bond environments.^65^ Note that the structural phase transition from β-FeOOH to ε-FeOOH should be accompanied by breaking and reforming the Fe–O bonds. This is because the *Pmn*2_1_ (ε-FeOOH) space group is a maximal nonisomorphic subgroup of the *Pnma* (α-FeOOH) space group, whereas *I*2/*m* or *I*4/*m* (ε-FeOOH) space group is not a minimal nonisomorphic supergroup of the *Pmn*2_1_ space group, based on the crystallographic relationship.^66^ Further HP/HT studies for various amounts of Cl^−^ ions and theoretical calculations would reveal detailed mechanisms of the structural phase transition from β-FeOOH and ε-FeOOH.

### 3.2 *x* and particle morphology

According to previous studies on β-FeOOH,^16,17,19,22,24–26^*x* in FeO_1−*x*_(OH)_1+*x*_Cl_*x*_ ranges from 0.003 to 0.37. Moreover, Cai *et al.*^16^ reported a decrease in *x* upon heating accompanying the structural phase transition to α-Fe_2_O_3_ (under ambient pressure). In this section, we discuss changes in *x* and the particle morphologies during the successive phase transitions of β-FeOOH → ε-FeOOH → α-Fe_2_O_3_ → Fe_3_O_4_. Only the composition of pristine β-FeOOH was analyzed, whereas TG was employed to analyze the HP/HT-treated samples because their weights were limited to *ca.* 40 mg.

The combined ICP-AES and IC analyses confirmed *x* = 0.12 for pristine β-FeOOH, suggesting the chemical formula FeO_0.88_(OH)_1.12_Cl_0.12_. The decomposition reaction upon heating is described by^26^

**Fig. 3:**
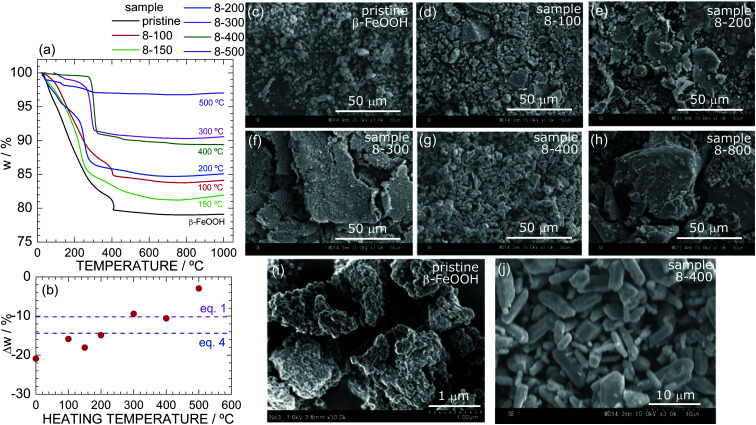
(a) TG curves of samples of pristine β-FeOOH, 8-100, 8-150, 8-200, 8-300, 8-400, and 8-500. (b) Δ*w* at 1000 °C as a function of the heating temperature during the HP/HT synthesis. The red and blue broken lines indicate the calculated Δ*w* based on eqn (1) and (4), respectively. SEM images at the 50 μm-scale of samples of (c) pristine β-FeOOH, (d) 8-100, (e) 8-200, (f) 8-300, (g) 8-400, and (h) 8-800. Enlarged SEM images of pristine β-FeOOH and 8-400 are shown in (i) and (j), respectively.

4 2FeO_0.88_(OH)_1.12_Cl_0.12_ → Fe_2_O_3_ + 0.24HCl↑ + H_2_O↑,

where Δ*w* is calculated to be −14.4%. However, as shown in Fig. 3a, Δ*w* of pristine β-FeOOH approaches −21% at 1000 °C; specifically, the weight (*w*) gradually decreases until a slight drop at *ca.* 400 °C and then remains at 79% up to 1000 °C. The discrepancy between the observed and calculated Δ*w* values is attributed to adsorbed water, residual ethylenediamine, and crystalline water in the sample. The gradual decrease in *w* and/or relatively large Δ*w* were also observed in previous thermal studies on β-FeOOH.^1,16,21,26^

The TG curve of sample 8-100 is similar to that of pristine β-FeOOH except for the Δ*w* value (=−15.9%), whereas the curves of samples 8-150, 8-200, 8-300, and 8-400 show different temperatures at which *w* rapidly decreases. For instance, sample 8-400 exhibits a rapid decrease in *w* at *ca.* 300 °C, which is *ca.* 100 °C lower than those of pristine β-FeOOH and sample 8-100. Fig. 3b reveals an almost linear relationship between Δ*w* at 1000 °C and the heating temperature during the HP/HT synthesis. Because the Δ*w* values of samples 8-300 and 8-400 are close to the calculated Δ*w* value (=10.14%) based on eqn (1) (Fig. 3b), the ε-FeOOH lattice is likely to be free from Cl^−^ ions. In other words, above 150 °C, the Cl^−^ ions start to evaporate out of the β-FeOOH lattice, which is associated with the structural phase transition to ε-FeOOH. The disappearance of Cl^−^ ions is strongly related to the mechanism underlying the structural phase transition; *i.e.*, as described above, it inevitably induces the breaking and reforming of Fe–O bonds.

We confirmed the absence of Cl^−^ ions in the ε-FeOOH lattice by comparing its TG curve with that of sample α-10-400 (Fig. S8†). Furthermore, after the TG analyses, we performed XRD and verified that pristine β-FeOOH and samples 8-100, 8-150, 8-200, 8-300, 8-400, 8-500, and α-10-400 all crystallized into the α-Fe_2_O_3_ phase (Fig. S9†). According to these results, the successive phase transitions of β-FeO_0.88_(OH)_1.12_Cl_0.12_ (at 8 GPa) are represented by

5 2β-FeO_0.88_(OH)_1.12_Cl_0.12_ → 2ε-FeOOH + 0.24HCl↑

below 400 °C and then

6 2ε-FeOOH → α-Fe_2_O_3_ + H_2_O↑

above 400 °C. The formation of Fe_3_O_4_ above 800 °C is described by eqn (2). The residual Cl^−^ ions on the surface of ε-FeOOH particles (sample 8-400) is discussed later.

Fig. 3c–h show SEM images of samples (c) pristine β-FeOOH, (d) 8-100, (e) 8-200, (f) 8-300, (g) 8-400, and (h) 8-800, which clarify changes in the particle morphologies and sizes during the phase transitions. Enlarged SEM images of pristine β-FeOOH and sample 8-400 are also shown in Fig. 3i and j, respectively. Particles in pristine β-FeOOH aggregate into secondary particles with an average size of 1 μm. As the heating temperature increases, secondary particles take on a scaly shape and form large secondary particles. Sample 8-400, which crystallized into the ε-FeOOH phase, shows a plate-like shape with an average size of 2 μm. Finally, sample 8-800, which crystallized into the Fe_3_O_4_ phase, exhibits a non-uniform shape with an average size of 50 μm. These changes indicate that the structural phase transitions under HP cause aggregation and change the particle size. Similar results were also observed for the phase transitions from α-FeOOH to ε-FeOOH^54^ and from spinel-structured Li[Li_1/3_Ti_5/3_]O_4_ to columbite-structured TiO_2_.^67^

We next investigated the effects of the applied pressure. Fig. S10† shows the SEM images of samples 6-200, 8-200, and 12-200, all of which were synthesized at 200 °C and crystallized into the ε-FeOOH phase. Fig. S11† shows the SEM images of samples 12-200, 12-400, 12-500, 12-700, which were synthesized at 12 GPa. The secondary particles of sample 6-200 indicate the same scaly shape as for sample 8-200, whereas those of sample 12-200 are relatively spherical. In addition, the primary particles of samples 12-400 and 12-500 are isolated from each other and are spherical with an average diameter of 3 μm. Thus, applying higher pressures gives the particles a round shape, although the samples are in the same crystalline phase. Fig. S12† shows the particle distribution of (a) β-FeOOH, (b) 8-400, and (c) 12-500 determined by the ImageJ software.^61^ The *d*_ave_ values of β-FeOOH, 8-400, and 12-500 were estimated to be 0.14, 4.7, and 2.9 μm, respectively.

### 3.3 OER activity

**Fig. 4:**
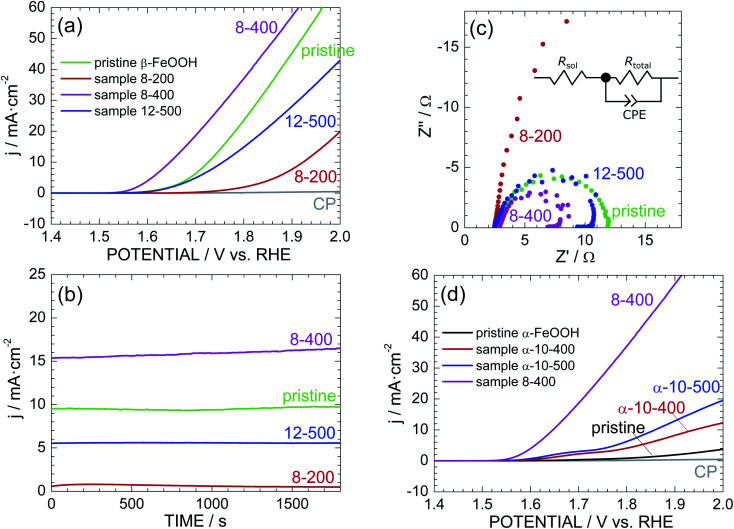
(a) LSV curves of pristine β-FeOOH and samples 8-200, 8-400, and 12-500. (b) Corresponding time courses of the anodic current measured at +1.70 V *vs.* RHE. (c) Cole–Cole plots of pristine β-FeOOH and samples 8-200, 8-400, and 12-500. Inset: equivalent circuit model consisting of *R*_sol_, *R*_total_, and CPE. (d) LSV curves of pristine α-FeOOH and samples α-10-400 and α-10-500 together with that of sample 8-400.

Fig. 4a shows the LSV curves of pristine β-FeOOH and samples 8-200, 8-400, and 12-500. The current density (*j*) was normalized by the total surface area of the electrode (2 cm^2^), while the potential *vs.* Ag/AgCl was converted into a potential *vs.* reversible hydrogen electrode (RHE). The anodic current of pristine β-FeOOH rapidly increases at *ca.* 1.60 V, which is consumed for the water oxidation accompanying with oxygen evolution.^3,4^ Sample 8-400 obviously exhibits a lower OER overpotential than that of pristine β-FeOOH; specifically, the anodic current rapidly increases above 1.55 V, in which threshold potential for OER is negatively shifted by *ca.* 60 mV compared to that of pristine β-FeOOH. For instance, the potential at which *j* = 1 mA cm^−2^ (*E*_*j*=1 mA cm^−2^_) was 1.62 V for pristine β-FeOOH and 1.56 V for 8-400. Sample 12-500 indicates a similar threshold potential to pristine β-FeOOH, whereas sample 8-200 shows a higher threshold potential (*ca.* 1.75 V). The *E*_*j*=1 mA cm^−2^_ values are summarized in Table 1. As evidenced by the XRD patterns shown in Fig. 2b, f, and S6,† samples 8-200, 8-400, and 12-500 crystallized into the ε-FeOOH phase. This suggests that OER activities of ε-FeOOH depend on synthesis conditions.

Fig. 4b shows the time courses of the anodic current of pristine β-FeOOH and samples 8-200, 8-400, and 12-500, measured at 1.70 V *vs.* RHE. Sample 8-400 still maintains the highest anodic current density of *ca.* 15 mA cm^−2^, which is *ca.* 1.5 times greater than that of pristine β-FeOOH. To reveal the origins of this OER activity, Fig. 4c compares the Cole–Cole plots of pristine β-FeOOH and samples 8-200, 8-400, and 12-500. Each sample exhibits one deformed semicircle, except for sample 8-200. EIS spectra generally include several contributions originated from the bulk, grain boundary, and surface of the electrode. However, we can simply estimate the total resistance (*R*_total_) of the electrode by the radius of the semicircle. The radius of sample 8-400 is apparently smaller than that of pristine β-FeOOH, indicating the decrease in *R*_total_ in the case for sample 8-400. More specifically, the EIS spectra were fitted to the equivalent circuit model shown in the inset of Fig. 4c, *i.e.*, a solution resistance (*R*_sol_), *R*_total_, and a constant-phase element (CPE). Here, the CPE is represented by 1/(*iwQ*)^*α*^; when *α* = 1, *Q* has the dimension of capacitance (F); *α* < 1 indicates surface heterogeneity in the system, such as the roughness of the electrode. As summarized in Table S1,†*R*_total_ = 9.42 and = 5.11 Ω for pristine β-FeOOH and sample 8-400, respectively, which suggests that one cause for enhancing OER activity of sample 8-400 is due to its lower *R*_total_.

As reported previously,^39–41^ α-FeOOH was also transformed into ε-FeOOH under HP/HT conditions. We thus examined the OER activity of pristine α-FeOOH and samples α-10-400 and α-10-500 for comparison. Here, samples α-10-400 and α-10-500, which crystallized into the ε-FeOOH phase. Fig. 4d reveals that the OER activity was enhanced by the phase transition from α-FeOOH to ε-FeOOH. However, the threshold potentials of samples α-10-400 and α-10-500 are much higher than those of samples 8-400 and 12-500, indicating the superiority of β-FeOOH over α-FeOOH. In other words, the OER activity of ε-FeOOH depends on the FeOOH source employed. The *E*_*j*=1 mA cm^−2^_ values of pristine α-FeOOH and samples α-10-400 and α-10-500 are listed in Table 1.

If we focus on the highest OER activity, this activity was enhanced in the order by α-FeOOH → β-FeOOH → ε-FeOOH. We next investigated the reproducibility of the OER activity among different electrodes prepared with the same sample. Fig. S13† shows the additional LSV curves of pristine β-FeOOH and sample 8-400. The LSV curves of the three different electrodes fabricated using pristine β-FeOOH appear similar, whereas those of the five different electrodes from sample 8-400 vary widely. Specifically, three of the five electrodes indicate superior OER activities regarding the threshold potential and anodic current density, whereas the rest show similar or lower OER activity to pristine β-FeOOH. Each electrode was prepared by depositing the sample into CP (Fig. 1f), so the OER activity probably depends on the distribution of particles and adhesion between the particles and CP.

### 3.4 Further characterization of sample 8-400

**Fig. 5:**
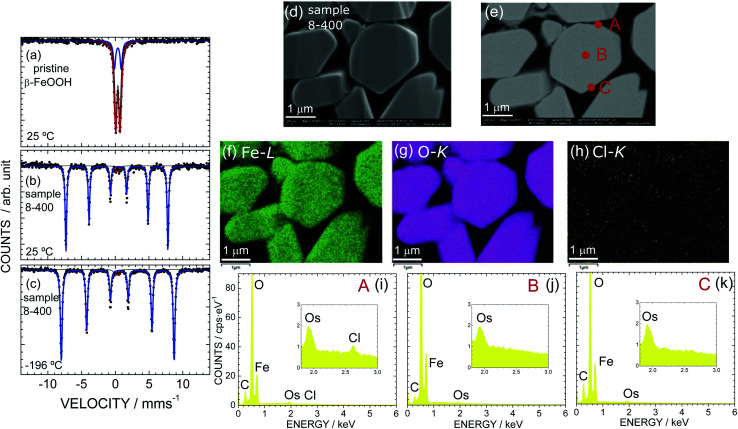
Mössbauer spectra of (a) pristine β-FeOOH at *ca.* 25 °C and sample 8-400 at *ca.* (b) 25 °C and (c) −196 °C recorded without applying a magnetic field. Red and blue lines indicate different magnetic components in the samples. (d) FE-SEM and (e) BSE images of sample 8-400. EDX mapping for (f) Fe, (g) O, and (h) Cl atoms and EDX spectra at points (i) A, (j) B, and (k) C in (e).

As sample 8-400 exhibited the best OER activity, we further characterized sample 8-400 using Mössbauer spectroscopy and EDX analysis. Fig. 5 shows the Mössbauer spectra of (a) pristine β-FeOOH at 25 °C and sample 8-400 at (b) 25 °C and (c) − 196 °C. A doublet peak appears for pristine β-FeOOH, indicating that it is in a paramagnetic state. The Mössbauer spectrum is ideally represented by7
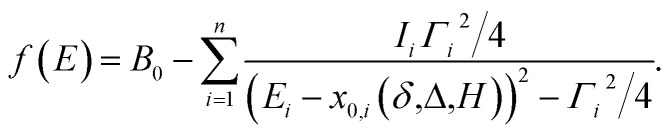
where *f*(*E*) and *B*_0_ are the counts at the Doppler velocity (*E*) and the baseline count, respectively; *I*_*i*_, *Γ*_*i*_, and *x*_0,*i*_ are the intensity, full-width at the half-maximum, and peak center at the *i*th peak, respectively; *δ* is the isomer shift; *Δ* is the quadruple splitting; and *H* is the internal field. The doublet peak consists of two components (*i* = 1 and 2), which are consistent with the two Fe sites, in the monoclinic symmetry. Table 4 lists the obtained Mössbauer parameters, namely, *δ*, *Δ*, *Γ*, *H*, and the component ratio. The Mössbauer parameters are comparable with previous results for β-FeOOH,^24^ although the origin of the two components was not fully understood in that investigation because the symmetry was wrongly concluded to be tetragonal.

**Table 4:** Mössbauer parameters of pristine β-FeOOH and sample 8-400

Sample	Component (*i*)	*δ* (mm s^−1^)	*Δ* (mm s^−1^)	*Γ* (mm s^−1^)	*H* (T)	Component ratio (%)
Pristine β-FeOOH (*ca.* 25 °C)	1	0.37	1.00	0.40	—	28
	2	0.37	0.58	0.40	—	72
8-400 (*ca.* 25 °C)	1	0.37	−0.24	0.26	46.9	97
	2	0.31	0.37	0.35	—	3
8-400 (−196 °C)	1	0.47	−0.24	0.31	51.8	97
	2	0.18	0.54	0.45	—	3

Sample 8-400 exhibits sextet peaks at both 25 and −196 °C, indicating that the sample is in an (antiferro)magnetic state (Fig. 5b and c). A careful analysis revealed that the Mössbauer spectra contain a small (3%) a paramagnetic contribution, as indicated by the red line in Fig. 5b and c. Considering each Mössbauer parameter summarized in Table 4 suggests that the paramagnetic contribution originates from the β-FeOOH phase. On the other hand, the Mössbauer parameters of the major contribution (97%) are determined to be *δ* = 0.37 mm s^−1^, *Δ* = −0.24 mm s^−1^, *Γ* = 0.26 mm s^−1^, and *H* = 46.9 T at *ca.* 25 °C. Because these Mössbauer parameters are similar to previous results for ε-FeOOH (*δ* = 0.15 mm s^−1^, *Δ* = −0.26 mm s^−1^, and *H* = 47.2 T at 23 °C),^68^ the major contribution is attributed to the ε-FeOOH phase. This mixed nature of sample 8-400 differs from the Rietveld results shown in Fig. S2† and Table 2, which suggest that the sample crystallizes into a single phase of ε-FeOOH. Note that sample α-10-400 comprises only from the ε-FeOOH phase, as understood by its Mössbauer spectrum in Fig. S14.†

According to the TG analyses (Fig. 3a and b), sample 8-400 was free from Cl^−^ ions in the ε-FeOOH lattice. However, we further investigated the distribution of Cl^−^ ions, particularly on the surface. Fig. 5d and e show the FE-SEM and BSE images of sample 8-400, respectively, while Fig. 5f, g, and h show the corresponding EDX mappings of Fe, O, and Cl atoms, respectively. Fe and O atoms are homogeneously distributed in the particles, whereas Cl atoms rarely appear. Based on the EDX spectra at points A, B, and C, Cl atoms are only observed at point A (Fig. 5i–k). Hence, the Cl^−^ ions are inhomogeneously distributed on a specific particle after the phase transition from β-FeOOH to ε-FeOOH. By contrast, we confirmed that the Cl^−^ ions are homogeneously distributed in the particles of pristine β-FeOOH, as shown in Fig. S15.† We also confirmed that there is no obvious change in the distribution of Fe and O atoms before and after the OER test (Fig. S16†).

### 3.5 Possible origin for high OER activity of ε-FeOOH

Considering the *d*_ave_ values of sample 8-400 (=4.7 μm) and pristine β-FeOOH (= 0.14 μm), the active surface area of sample 8-400 should be smaller than that of pristine β-FeOOH. This indicates that the OER activity of sample 8-400 is attributed to an intrinsic and high OER performance of ε-FeOOH. In other words, a small *R*_bulk_ contributes to the relatively low *R*_total_ of sample 8-400 (= 5.11 Ω).

Based on studies of quadruple manganese perovskites AMn_7_O_12_ (A = La and Ca) which were synthesized by the HP/HT method,^69^ their OER activities relate with the nearest bond distance between O atoms (*d*_O–O_); the overpotential decreases with *d*_O–O_. Indeed, as shown in Fig. S17,† the *d*_O–O_ value was decreased in the order by α-FeOOH (=3.035 Å) → β-FeOOH (=3.023 Å) → ε-FeOOH (=3.006 Å). Furthermore, it is interesting to compare the OER activities of ε-FeOOH and β-MnO_2_ (pyrolusite), as they are almost isostructural.^27^ Yan *et al.* clarified that the phase transition from β-MnO_2_ to α-MnO_2_ (cryptomelane) improved the OER activity,^30^ which is the opposite of the trend observed in this study. Note that both β-MnO_2_ and ε-FeOOH are classified as rutile, although ε-FeOOH is regarded as a distorted rutile structure, as in the case of columbite-type TiO_2_.^67^ Thus, there is a possibility that the superior OER activity of ε-FeOOH owes to its characteristic structural environments such as the shortest *d*_O–O_ and slightly distorted FeO_6_ octahedra.

Another possibility would come from the mixed nature of sample 8-400. Hu *et al.*^6^ recently reported that using mixed phases of α- and β-FeOOH or β- and *δ*-FeOOH enhanced the OER activity owing to the formation of oxygen vacancies on the surface. As sample 8-400 contains a small amount (3%) of β-FeOOH, synergetic effects of the mixture of ε- and β-FeOOH could contribute to its OER activity.

Anyway, comparing the OER activities of samples 8-200 and 12-500 suggests that other factors besides the crystalline phase, such as the particle morphology and particle size, affect the OER activities. Further theoretical studies on FeOOH polymorphs would reveal details of the reaction mechanism underlying the OER activity of ε-FeOOH.

### 3.6 Perspective of ε-FeOOH as catalyst

We revealed that ε-FeOOH exhibited the best OER activity among the FeOOH polymorphs investigated. However, as summarized in Table 1, even the best OER activity of ε-FeOOH was restricted to *E*_*j*=1 mA cm^−2^_ = 1.56 V *vs.* RHE. This overpotential is still higher than those of other non-noble metal-based electrocatalysts; for instance, an exfoliated NiFe layered double hydroxide indicated *E*_*j*=1 mA cm^−2^_ ≃ 1.52 V *vs.* RHE.^72^ The relatively large overpotential of ε-FeOOH is caused by its large particle size (*d*_ave_ > 2 μm), namely low active site for OER, as shown in Fig. 3j and S12.† The procedure for preparing composite electrodes also results in the large overpotential, because the composite electrode with the colloridal β-FeOOH solution showed a lower (*ca.* 0.05 V) overpotential.^3–5,48^ Therefore, investigations for decreasing particle size and optimizing composite electrodes could enhance OER activity of ε-FeOOH. The pressure-phase diagram shown in Fig. 2l would be useful for these investigations. Finally, ε-FeOOH is applicable as a water oxidation photocatalyst,^70,71^ although the present study is focused on the electrocatalytic properties.

## 4 Conclusion

We examined HP/HT phases of β-FeOOH nanorods, more specifically, β-FeO_1−*x*_(OH)_1+*x*_Cl_*x*_ with *x* = 0.12, and their OER activities. To our best knowledge, we firstly demonstrated that β-FeOOH transformed into ε-FeOOH above 6 GPa and 100 °C. In addition, Cl^−^ ions, which were initially hosted in the [2 × 2] tunnels, evaporated from the surface during the structural phase transition. Above 400 °C, ε-FeOOH decomposed into α-Fe_2_O_3_ or Fe_3_O_4_, while releasing oxygen from the lattice. Above 6 GPa, ε-FeOOH was stabilized at higher temperatures; for instance, at 12 GPa, ε-FeOOH decomposed into α-Fe_2_O_3_ above 700 °C. The obtained pressure–temperature phase diagram of β-FeOOH differed from that of α-FeOOH, probably owing to the open framework of β-FeOOH and partial occupation of Cl^−^ ions. In the viewpoint from earth and space sciences, this information is crucial for in-depth understanding of evolution and dynamics of the Earth interior particularly at the upper mantle.

Sample 8-400 (ε-FeOOH), which was synthesized at 8 GPa and 400 °C, exhibited superior OER activity over the parent β-FeOOH or α-FeOOH. EIS results showed that this superior OER activity is due to its low *R*_total_. Moreover, Mössbauer spectroscopy revealed that sample 8-400 contains a small amount (3%) of the β-FeOOH phase, possibly leading to synergetic effects from β- and ε-FeOOH. The residual Cl^−^ ions were inhomogeneously distributed on the surfaces of particles in sample 8-400, resulting in relatively scattered OER activities along different composite electrodes fabricated from this sample. Nonetheless, ε-FeOOH is a possible candidate as a next-generation earth-abundant OER catalyst, particularly because material options are currently limited. The established pressure–temperature phase diagram is useful for further improving the OER activity of ε-FeOOH, because particle sizes, particle morphologies, and distribution of Cl^−^ ions strongly depend on the HP/HT conditions.

## Conflicts of interest

There are no conflicts to declare.

## Supplementary Material

RA-010-D0RA09895G-s001
