# Supplementary material for: High-pressure synthesis of ε-FeOOH from β-FeOOH and its application to the water oxidation catalyst
Source: RSC Adv. 2020 Dec 18;10(73):44756–67. doi: 10.1039/d0ra09895g (PMC9058670; doi:10.1039/d0ra09895g)
Supplement: RA-010-D0RA09895G-s001 [file RA-010-D0RA09895G-s001.pdf]

Electronic Supplementary Material (ESI) for RSC Advances.
This journal is © The Royal Society of Chemistry 2020

Supplementary Information

High-pressure synthesis of ϵ-FeOOH from β-FeOOH and its application to the water oxidation catalyst

Kazuhiko Mukai,*,† Tomiko M. Suzuki,† Takeshi Uyama,† Takamasa Nonaka,† Takeshi Morikawa,† and Ikuya Yamada‡

Toyota Central Research and Development Laboratories, Inc., 41–1 Yokomichi, Nagakute, Aichi 480–1192, Japan, and Department of Materials Science, Graduate School of Engineering, Osaka Prefecture University, 1–2 Gakuen-cho, Sakai, Osaka 599–8570, Japan

*E-mail: e1089@mosk.tytlabs.co.jp

Phone: +81-561-71-7698. Fax: +81-561-63-6119

*To whom correspondence should be addressed

†Toyota Central Research and Development Laboratories, Inc., 41–1 Yokomichi, Nagakute, Aichi 480–1192, Japan

‡Department of Materials Science, Graduate School of Engineering, Osaka Prefecture University, 1–2 Gakuen-cho, Sakai, Osaka 599–8570, Japan

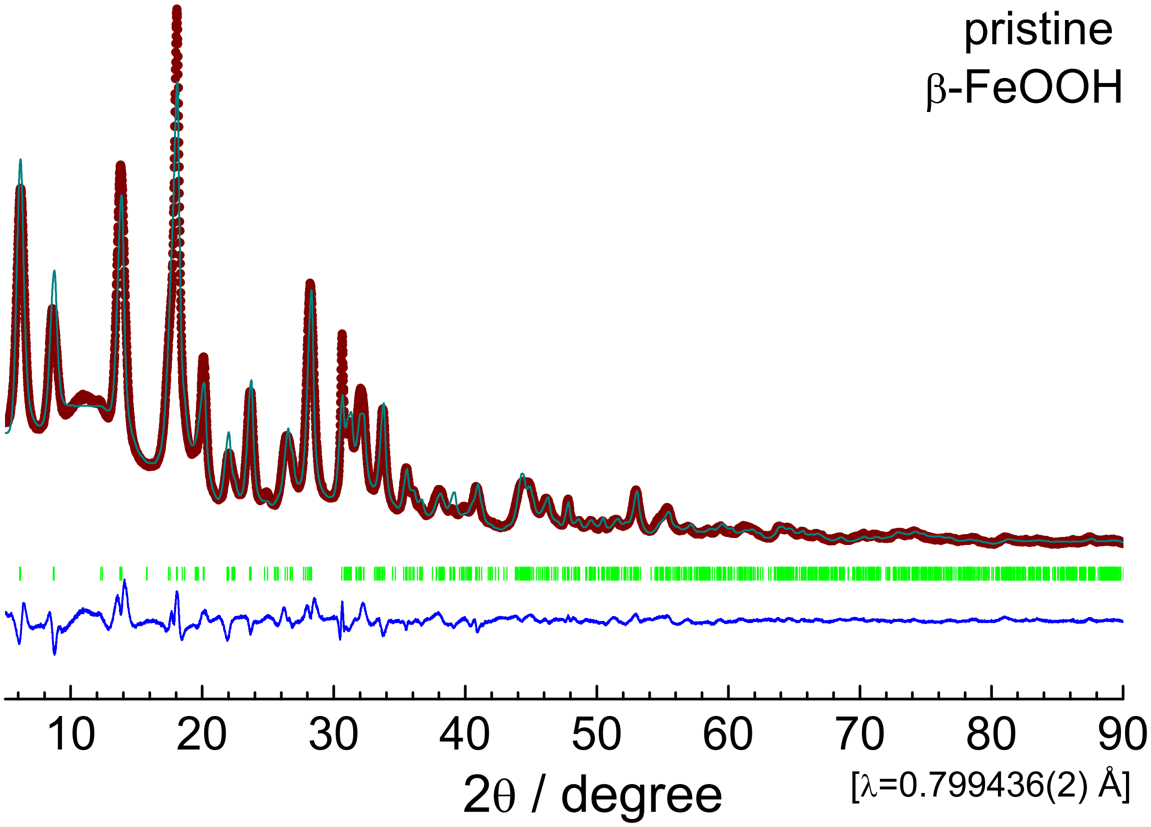

Fig. S1: Rietveld analysis result of pristine β-FeOOH. We employed the monoclinic symmetry with $I4/m$ space group by ignoring the presence of H^+ ions.

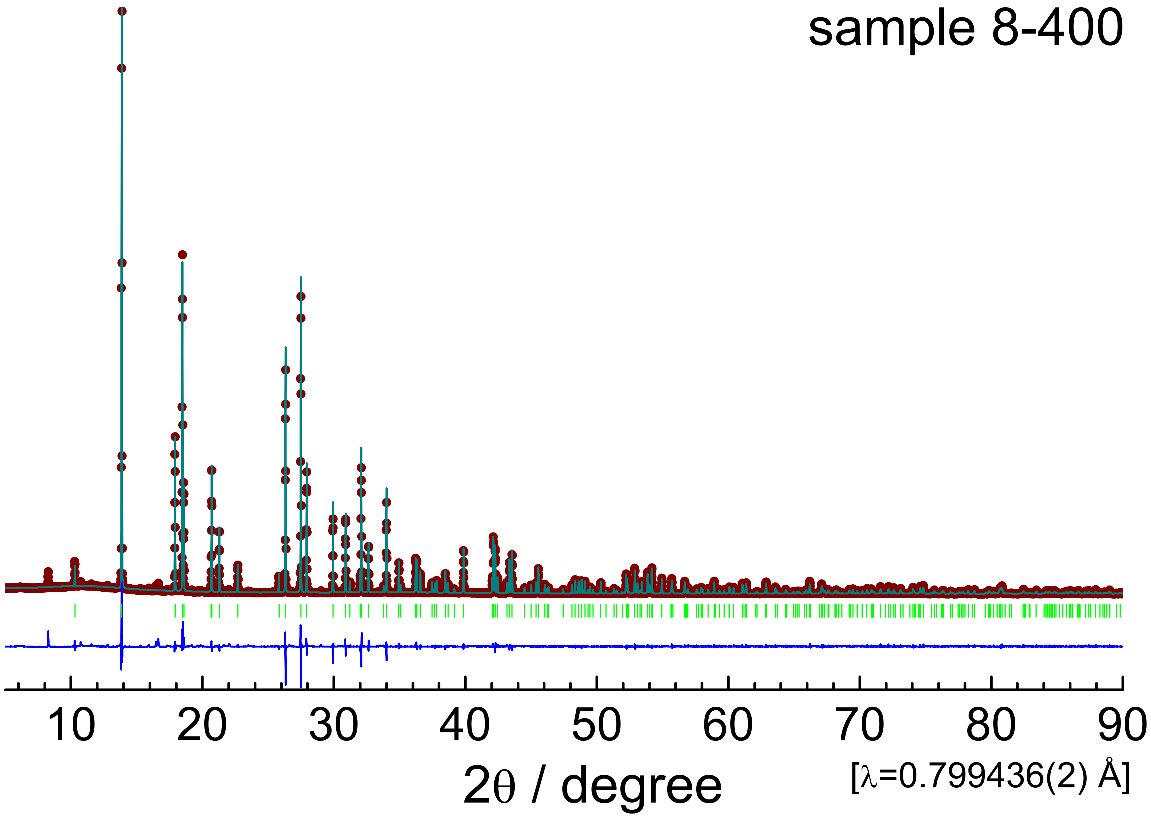

Fig. S2: Rietveld analysis result of sample 8-400, which possesses the ϵ-FeOOH-type structure with $Pmn2_1$ space group.

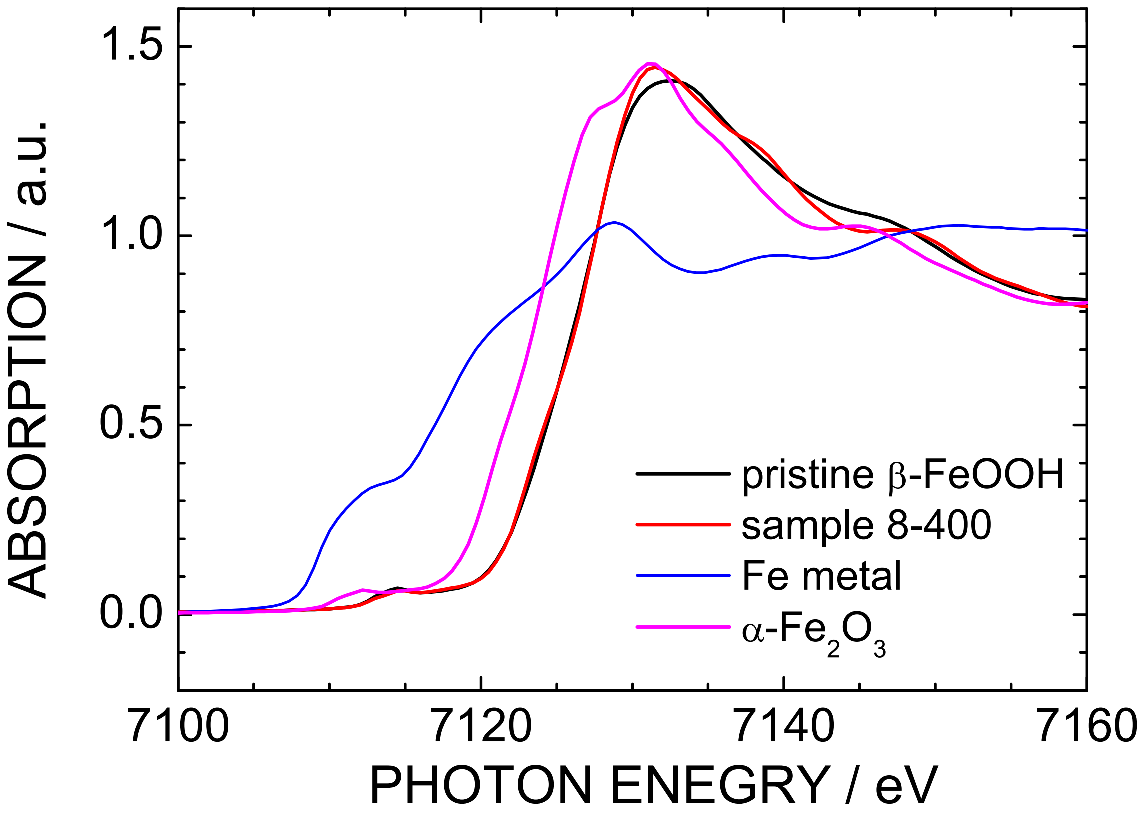

Fig. S3: Fe *K*-edge XANES spectra of Fe metal and α-Fe_2O_3 together with those of samples of pristine β-FeOOH and 8-400. The peak top energy was 7131.4 eV for α-Fe_2O_3 and 7128.7 eV for Fe metal.

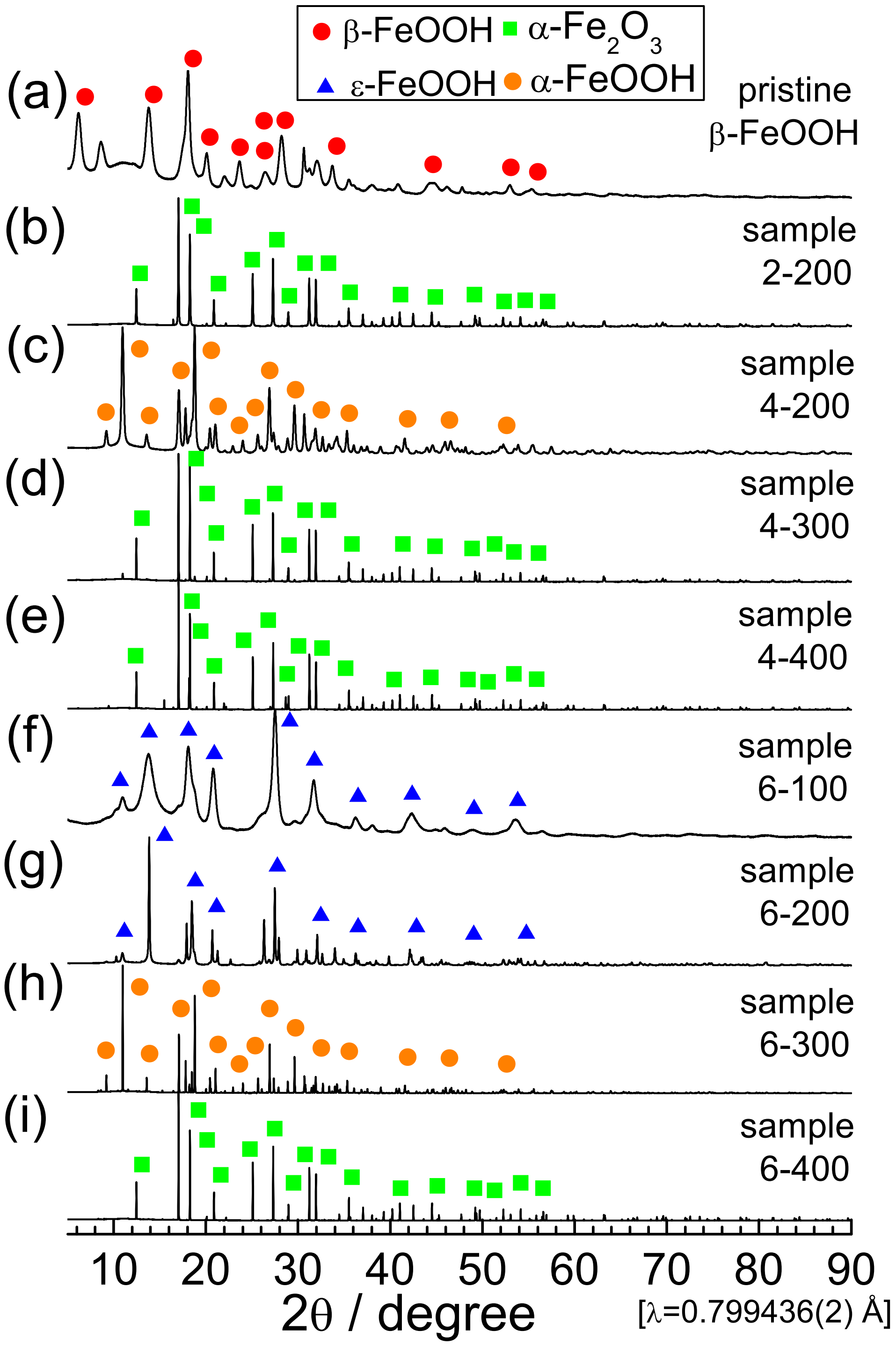

Fig. S4: Synchrotron XRD patterns of samples (a) pristine β-FeOOH, (b) 2-200, (c) 4-200, (d) 4-300, (e) 4-400, (f) 6-100, (g) 6-200, (h) 6-300, and (i) 6-400.

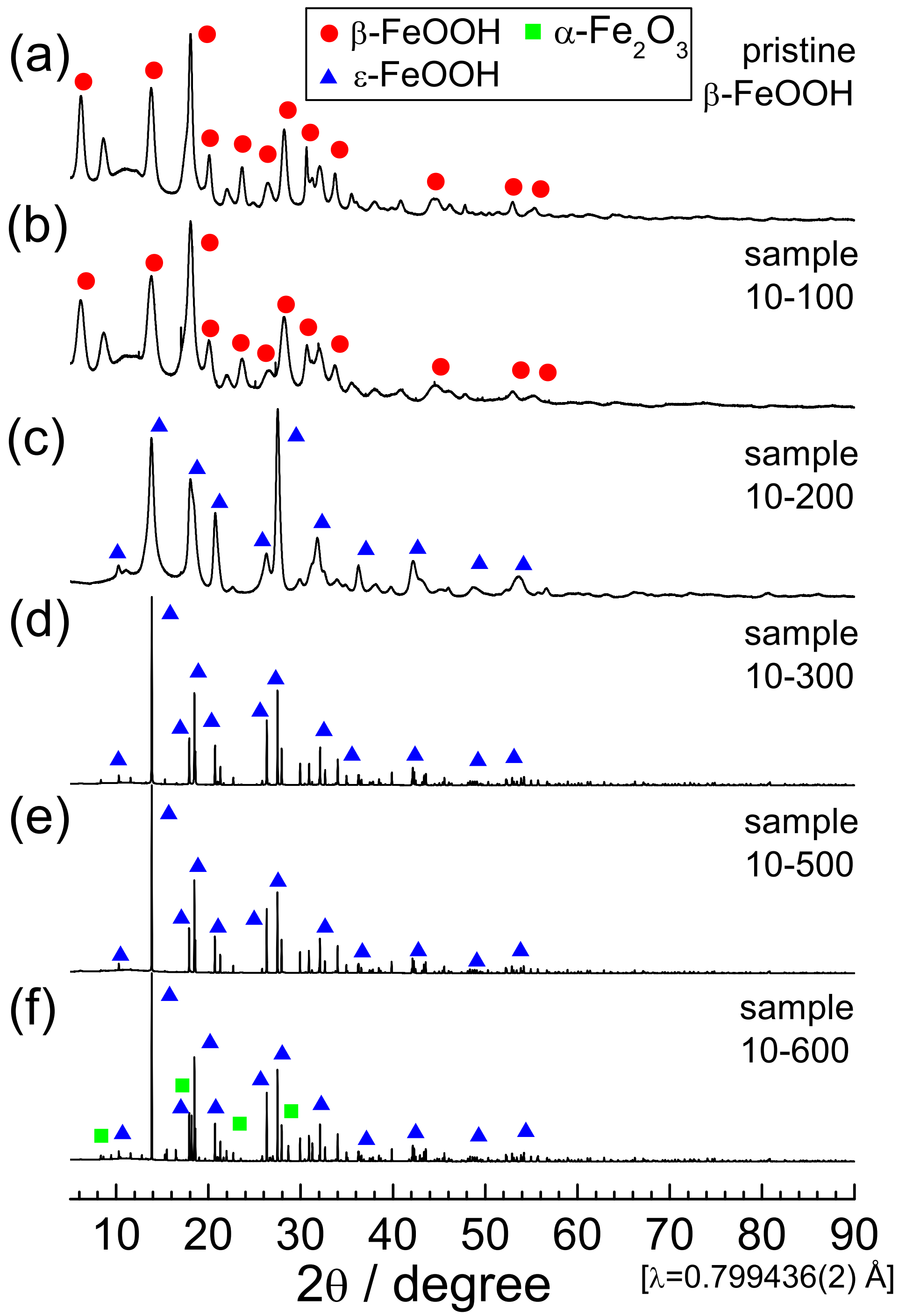

Fig. S5: Synchrotron XRD patterns of samples (a) pristine β-FeOOH, (b) 10-100, (c) 10-200, (d) 10-300, (e) 10-500, and (f) 10-600.

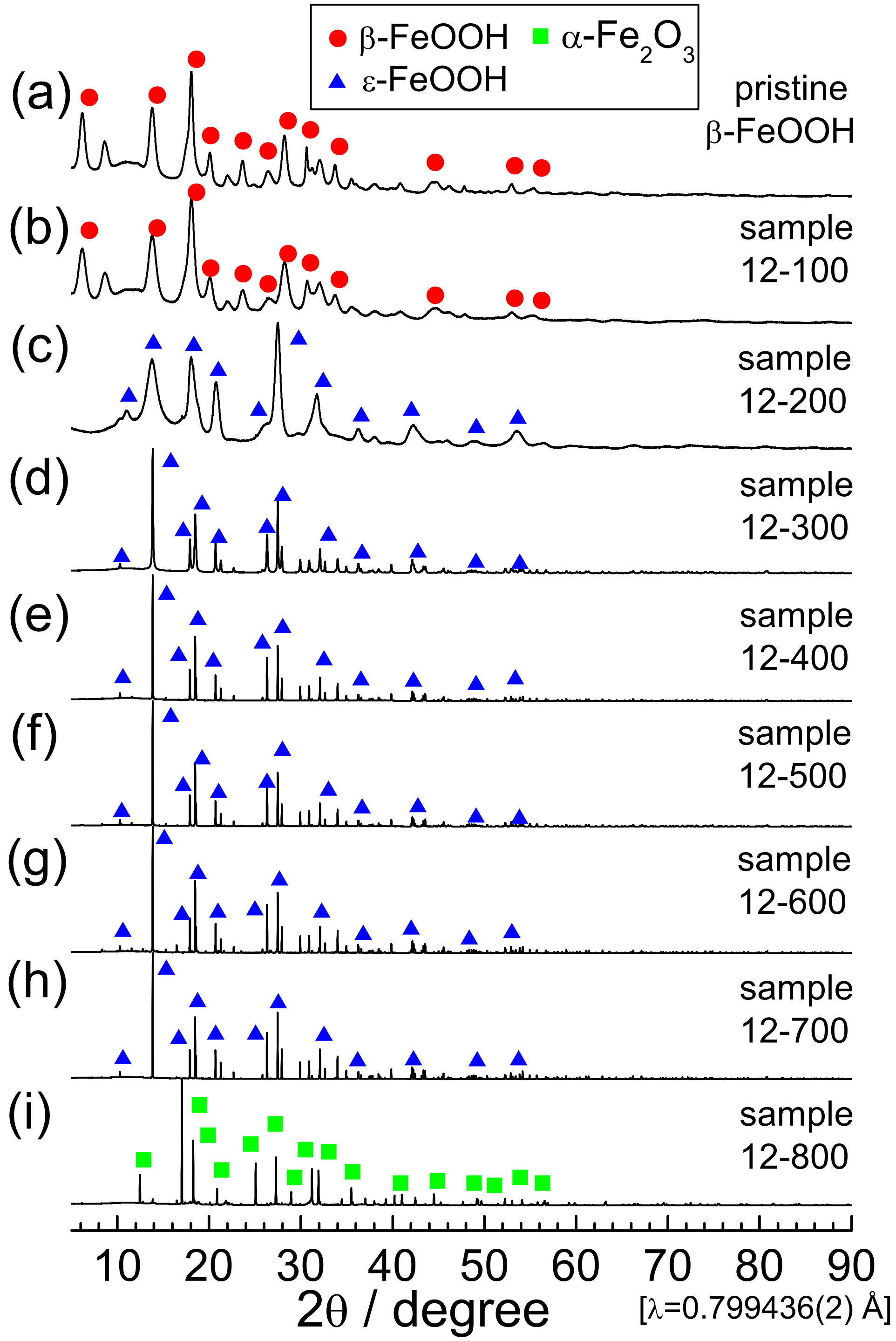

Fig. S6: Synchrotron XRD patterns of samples (a) pristine β-FeOOH, (b) 12-100, (c) 12-200, (d) 12-300, (e) 12-400, (f) 12-500, (g) 12-600, (h) 12-700, and (i) 12-800.

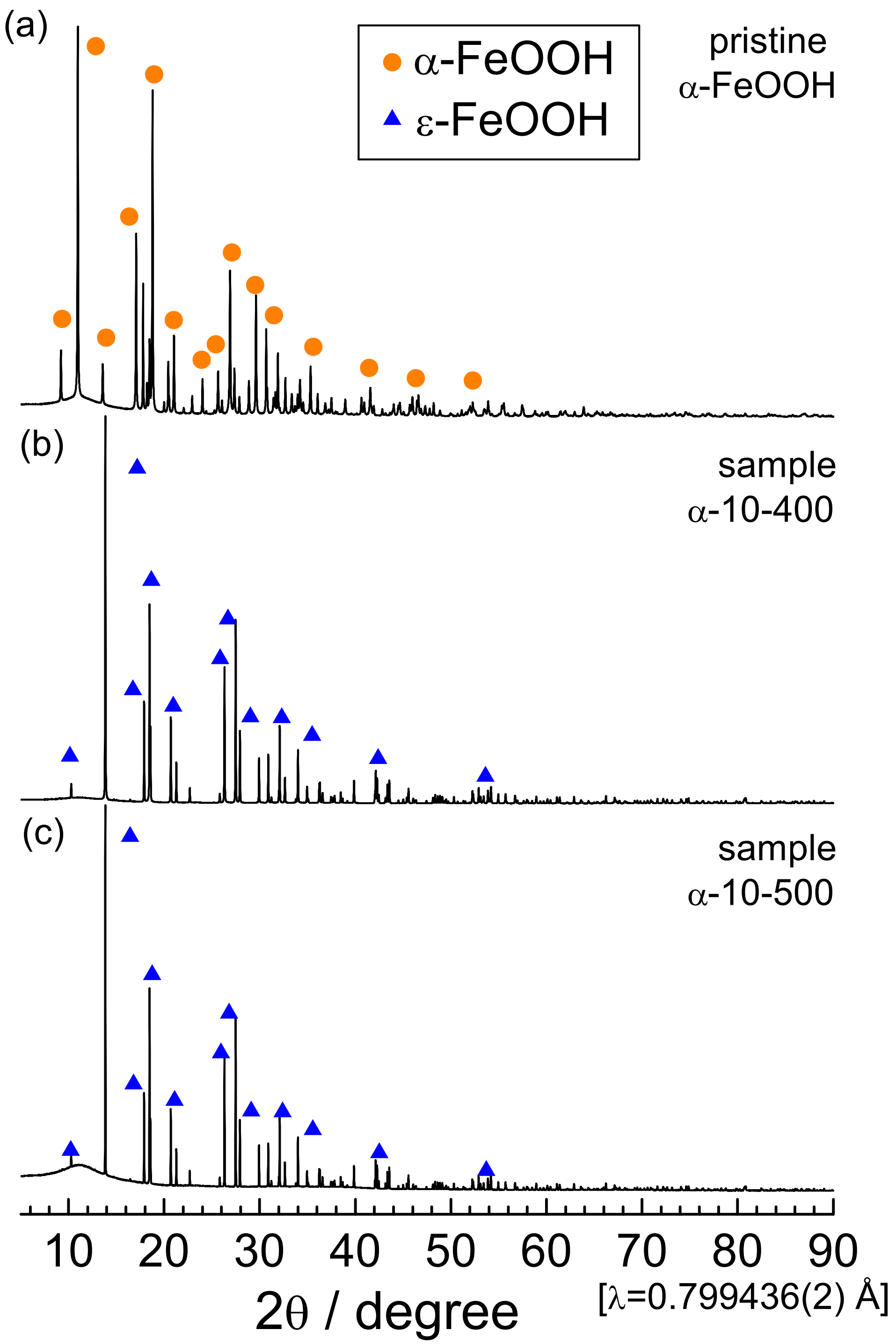

Fig. S7: Synchrotron XRD patterns of samples (a) pristine α-FeOOH, (b) α-10-400 and (c) α-10-500.

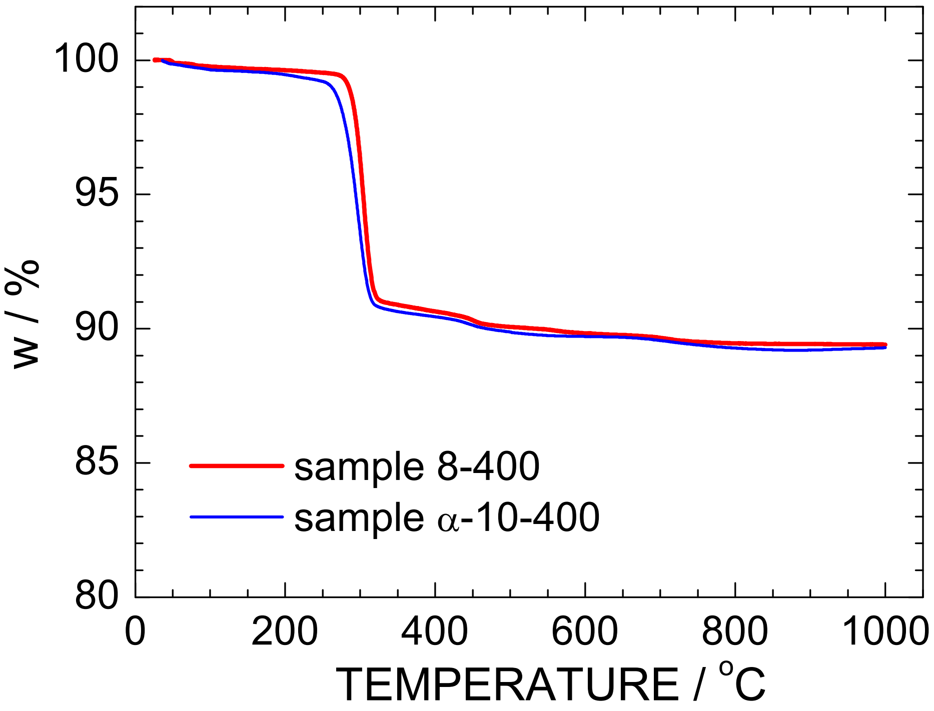

Fig. S8: Comparison of the TG curves of samples of 8-400 and α-10-400.

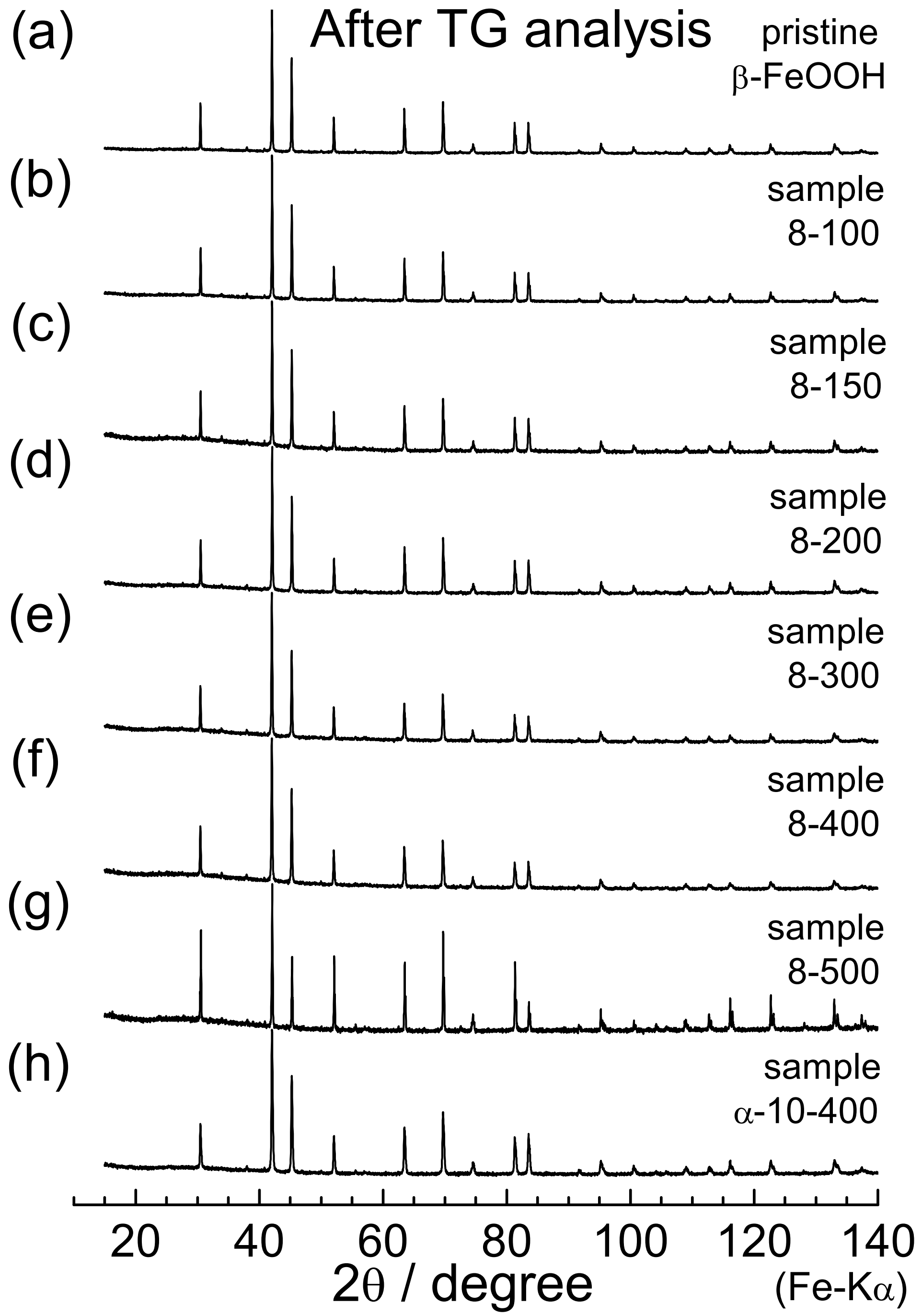

Fig. S9: XRD patterns after the TG analysis of samples (a) pristine β-FeOOH, (b) 8-100, (c) 8-150, (d) 8-200, (e) 8-300, (f) 8-400, (g) 8-500, and (h) α-10-400.

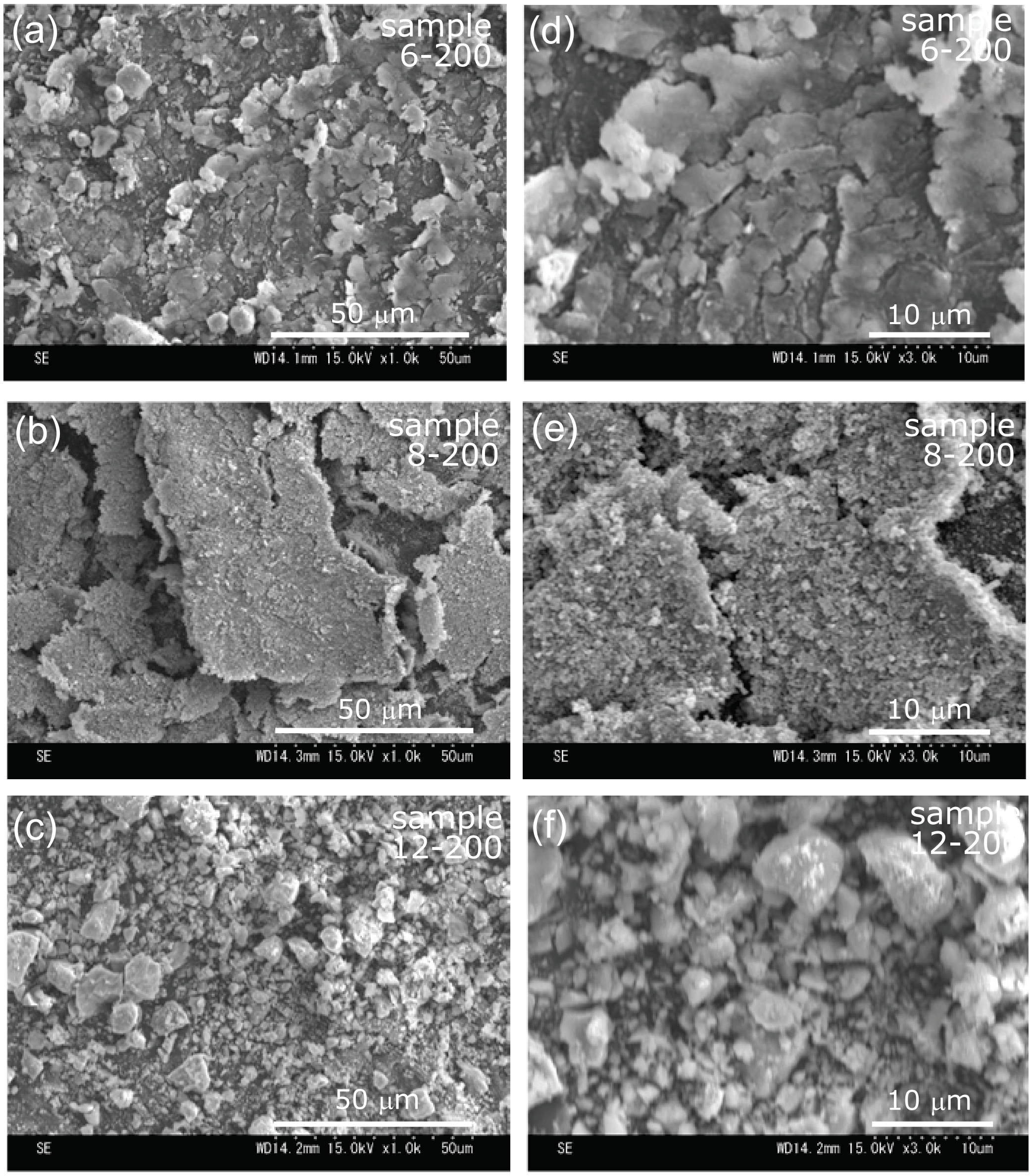

Fig. S10: SEM images at the 50μm-scale of samples (a) 6-200, (b) 8-200, and (c) 12-200. Corresponding enlarged SEM images are shown in (d)–(f).

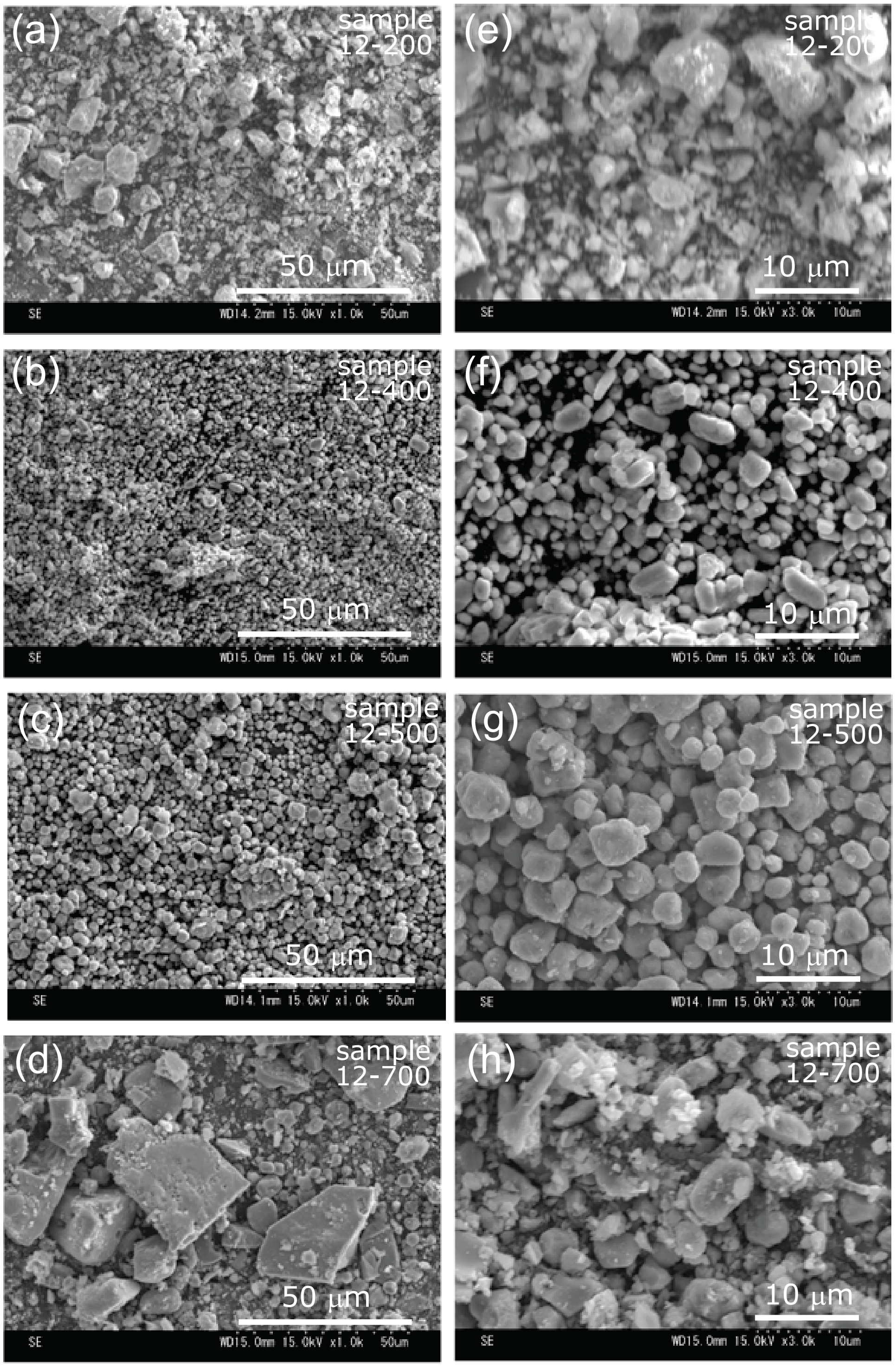

Fig. S11: SEM images at the 50μm-scale of samples (a) 12-200, (b) 12-400, (c) 12-500, and (d) 12-700. Corresponding enlarged SEM images are shown in (e)–(h).

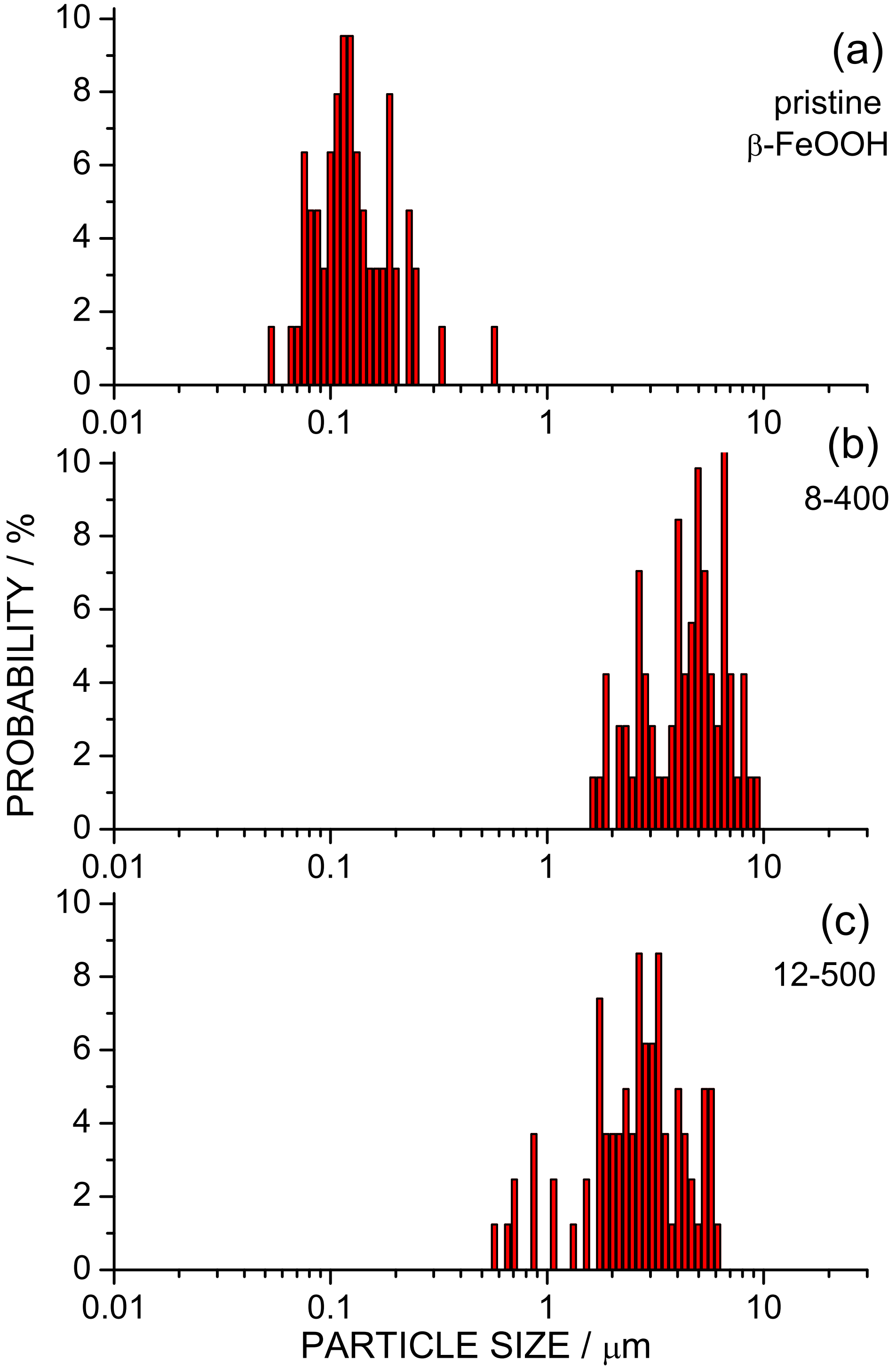

Fig. S12: Particle distribution of (a) pristine β-FeOOH, (b) 8-400, and (c) 12-500 determined by the Image J software.

Table S1: Fitting results of Cole-Cole plots for samples of pristine β-FeOOH, 8-200, 8-400, and 12-500

Sample	R_{sol} / Ω	R_{total} / Ω	CPE / F	α
pristine β-FeOOH	2.66	9.42	25.7×10^{-6}	0.915
8-200	2.52	91.1	3.47×10^{-6}	0.942
8-400	2.98	5.11	3.92×10^{-6}	1.02
12-500	2.57	8.50	7.44×10^{-6}	0.953

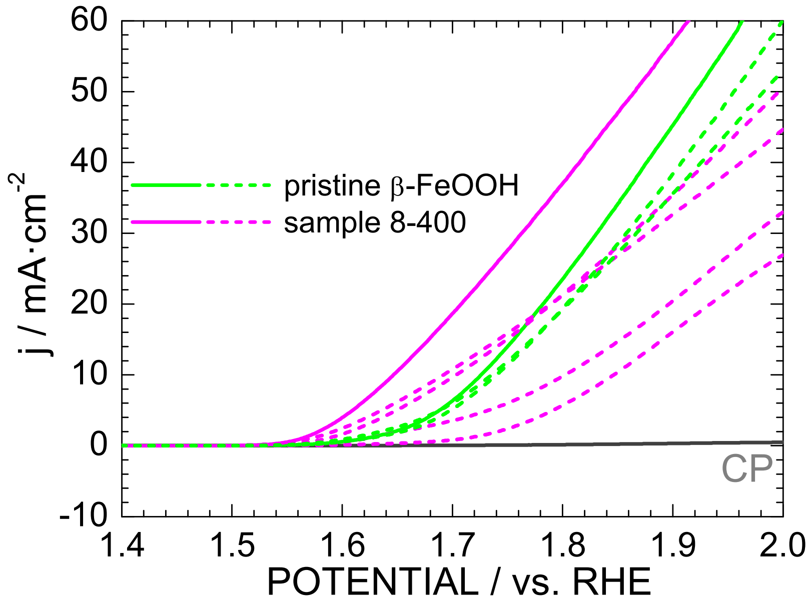

Fig. S13: Additional LSV curves of pristine β-FeOOH ans sample 8-400.

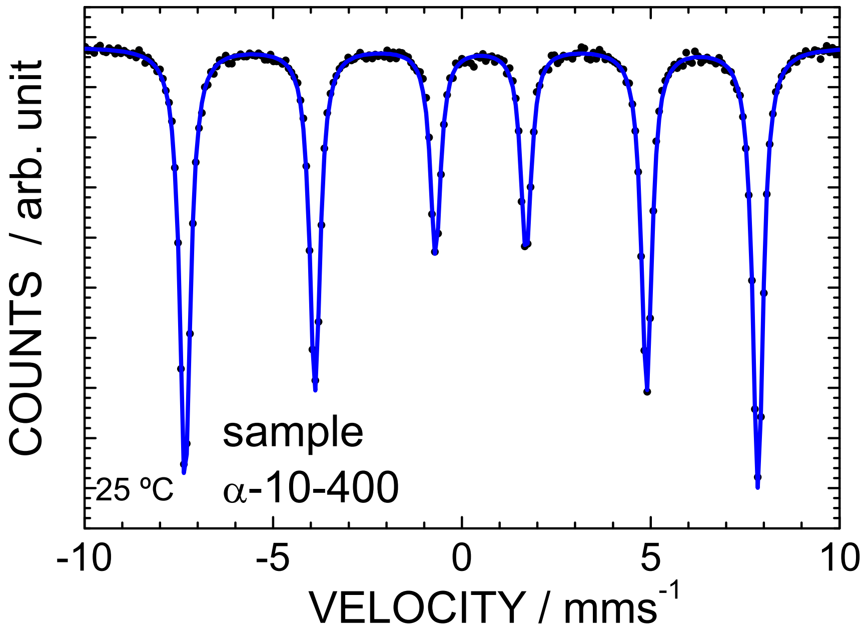

Fig. S14: Mössbauer spectrum of sample α-10-400 at ca. 25 °C.

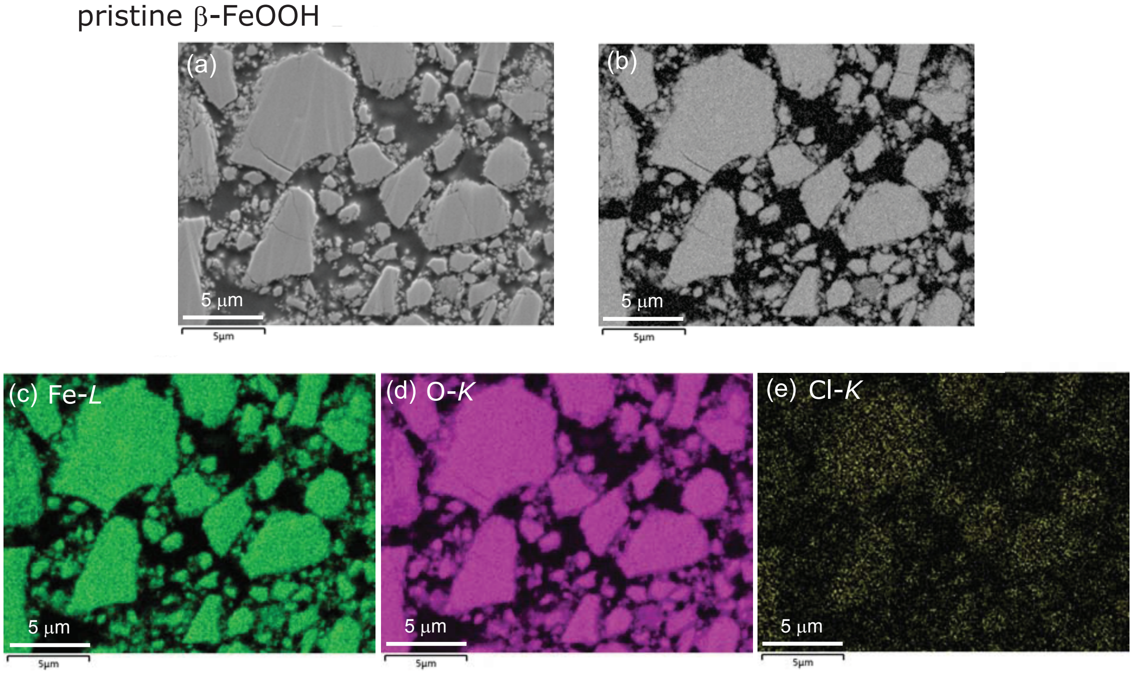

Fig. S15: SEM images of pristine β-FeOOH obtained by using (a) secondary electrons and (b) back scattered electrons. EDX mapping for (c) Fe, (d) O, and (e) Cl atoms.

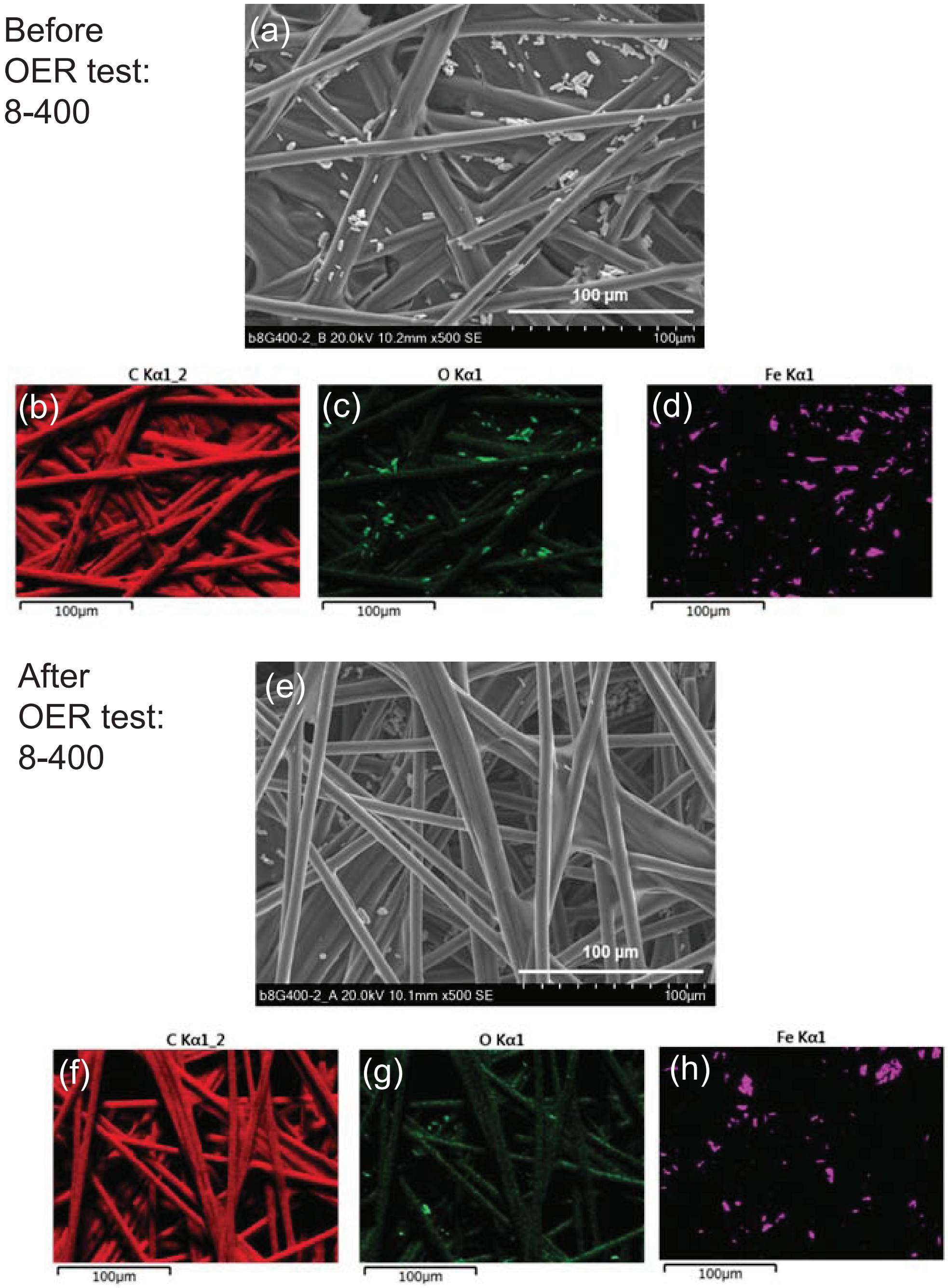

Fig. S16: SEM images and EDX mappings for sample 8-400: (a)–(d) before and (e)–(h) after.

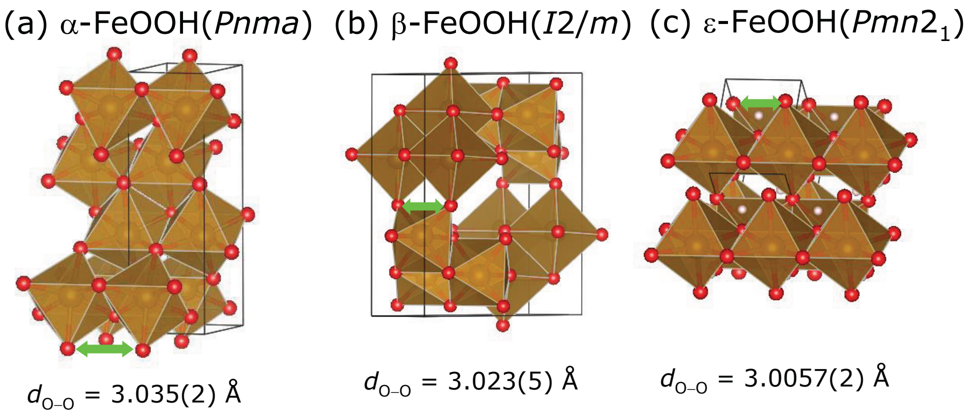

Fig. S17: Crystal structure and d_{O-O} of (a) α-FeOOH, (b) β-FeOOH, and (c) ϵ-FeOOH.
